# The impact of whole-molecule disorder on spin-crossover in a family of isomorphous molecular crystals†

**DOI:** 10.1039/d5sc00090d

**Published:** 2025-04-10

**Authors:** Holly E. Sephton, Rhiannon L. Watson, Namrah Shahid, Hari Babu Vasili, Daniel L. Baker, Dipankar Saha, Izar Capel Berdiell, Christopher M. Pask, Oscar Cespedes, Malcolm A. Halcrow

**Affiliations:** a School of Chemistry, University of Leeds Woodhouse Lane Leeds LS2 9JT UK m.a.halcrow@leeds.ac.uk; b School of Physics and Astronomy, University of Leeds W. H. Bragg Building Leeds LS2 9JT UK; c Department of Chemistry, Faculty of Mathematics and Natural Sciences, University of Oslo 0371 Oslo Norway; d Center for Material Science and Nanomaterials (SMN), University of Oslo 0371 Oslo Norway

## Abstract

Treatment of 2-(pyrazol-1-yl)-6-fluoropyridine with one equiv. of the appropriate 4-substituted 1*H*-pyrazole in the presence of sodium hydride gives moderate yields of 2-(pyrazol-1-yl)-6-(4-methylpyrazol-1-yl)pyridine (L^Me^), 2-(pyrazol-1-yl)-6-(4-fluoropyrazol-1-yl)pyridine (L^F^), 2-(pyrazol-1-yl)-6-(4-chloropyrazol-1-yl)pyridine (L^Cl^) and 2-(pyrazol-1-yl)-6-(4-bromopyrazol-1-yl)pyridine (L^Br^). Single crystals of [Fe(L^R^)_2_]Z_2_ (R = Me, F or Br; Z^−^ = BF_4_^−^ or ClO_4_^−^) are often well-formed, but are poor diffractors of X-rays. An analysis of [Fe(L^Me^)_2_][ClO_4_]_2_ showed non-statistical positional disorder of the methyl substituents, leading to whole molecule disorder in each residue of the asymmetric unit. Single crystals of [Fe(L^Br^)_2_][BF_4_]_2_ are isomorphous with the L^Me^ complex, but show less substituent disorder. All the complex salts are isomorphous by powder diffraction, and show thermal spin-transitions whose cooperativity differs from gradual (R = Me) to abrupt and hysteretic (R = Br). Some of the cooperative transitions exhibit irregular, closely spaced discontinuities which are not caused by crystallographic phase changes, and may reflect local heterogeneities associated with the cation disorder. No aspect of their crystal packing appears to correlate with their spin-transition cooperativity. However, weaker cooperativity may correlate with increased cation disorder in this system, which merits further investigation.

## Abstract 2

Seven [Fe(L^R^)_2_]^2+^ (L^R^ = 2-{pyrazolyl}-6-{4-R-pyrazolyl}pyridine) salts are isomorphous, with substituents disordered around the complex, but show a variety of thermal spin-transition behaviors.

## Graphical abstract

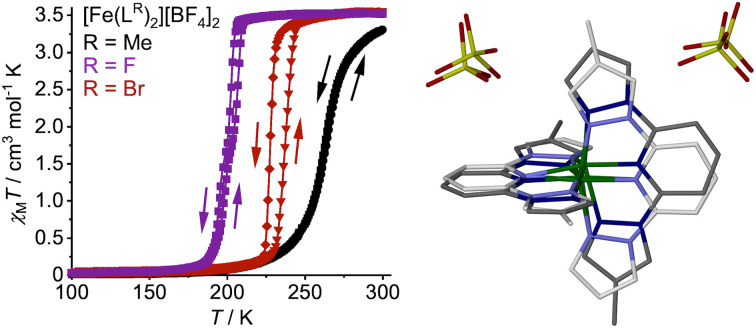

## Introduction

Spin-crossover (SCO) materials undergo a reversible spin state change which can be induced thermally,^1–6^ under irradiation^7–9^ or pressure,^10,11^ or with other physical stimuli.^8,12^ SCO compounds are versatile molecular switches at the molecular level, and as bulk materials. As such they have interest as switching components in multifunctional molecular materials;^13–17^ for nano- and micro-scale molecular electronics;^18–21^ and for macroscopic applications such as solid state refrigeration,^22–24^ thermochromic printing,^25,26^ optical switches^27^ and mechanical actuation devices.^28–30^ Most of these applications require materials exhibiting SCO over a desired temperature range, usually (but not always) with thermal hysteresis. Crystal engineering such properties into new materials is another important aspect of SCO research.^31–34^

**Scheme 1:**
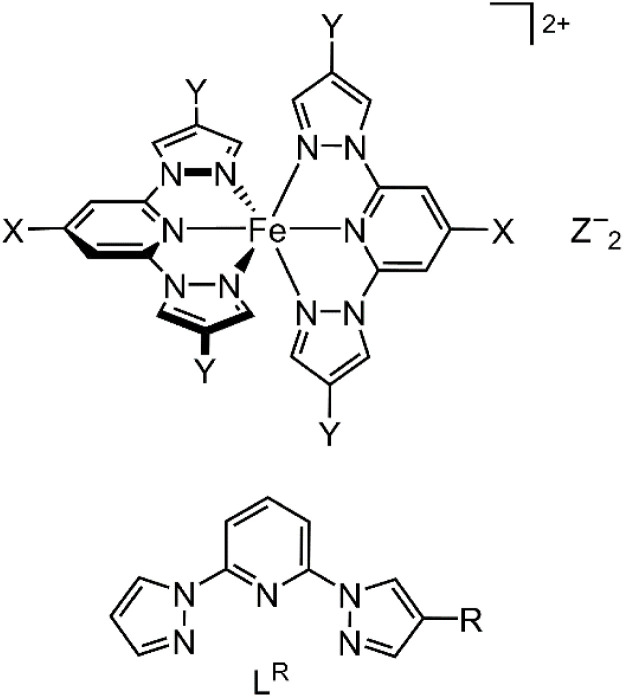
(Top) Structure of [Fe(bpp^X,Y^)_2_]Z_2_ (Z^−^ = a monovalent anion; the parent complex [Fe(bpp)_2_]^2+^ has X = Y = H). (Bottom) The new ligands L^R^ (R = Me, F, Cl, Br).

Iron(ii) complexes of 2,6-di(pyrazol-1-yl)pyridine (bpp) and its derivatives are some of the most studied SCO compounds.^35–37^ The attraction of bpp complexes for SCO research is their synthetic flexibility, which allows substituents to be appended to any position of the ligand framework. This has afforded [Fe(bpp^X,Y^)_2_]Z_2_ derivatives (Scheme 1) with functional ‘X’ substituents for multifunctional materials,^38–43^ or tether groups for nanoscience applications.^44–46^

The library of SCO compounds based on the same [Fe(bpp^X,Y^)_2_]Z_2_ motif is also a useful source of structure : function relationships for SCO materials design.^47–50^ The subset of compounds adopting the “terpyridine embrace” structure type^51^ has particular value in that regard. That structure is adopted by a number of [Fe(bpp^X,Y^)_2_]Z_2_ derivatives with small ligand substituents (X and Y = H, Me, halogen *etc.*). Most of these exhibit consistently abrupt spin-transitions with narrow thermal hysteresis,^47,49,52,53^ although exceptions showing more gradual SCO or discontinuous transitions also exist.^54–57^ The near-isomorphous nature of these compounds allows similarities and differences in their SCO behaviour to be correlated with specific features of their structural chemistry.^48,49,57,58^

The bpp ligand skeleton is usually synthesised from 2,6-dihalopyridine precursors, by nucleophilic substitution with deprotonated 1*H*-pyrazoles. The first reports of this procedure noted that the substitution steps can be performed sequentially using different pyrazolate reagents to give bpp derivatives with different substituents on each pyrazolyl ring.^59,60^ However, thus far few unsymmetric bpp derivatives have been investigated in SCO materials.^43,53,61^ We now report iron(ii) complexes of three new bpp derivatives L^R^, bearing a small peripheral ‘R’ substituent at the *C*4 position of one pyrazolyl ring (R = Me, F or Br; Scheme 1).^62^ All the complex salts exhibit thermal spin transitions below room temperature, with varying degrees of cooperativity. Structural characterisation of the complex salts was challenging, but has yielded further insights into the SCO characteristics of the terpyridine embrace crystal lattice.

## Results

Treatment of 2,6-difluoropyridine with 1 equiv. of 1*H*-pyrazole in dmf at room temperature, in the presence of sodium hydride, yields 2-(pyrazol-1-yl)-6-fluoropyridine in variable yields of 30–50%.^63,64^ Doubly substituted bpp is always a byproduct of this reaction, which must be removed by flash silica chromatography. The L^R^ ligands (R = Me, F, Cl or Br) were obtained by treating 2-(pyrazol-1-yl)-6-fluoropyridine with 1 equiv. of the appropriate 4-substituted 1*H*-pyrazole, under the same conditions as before. The unsymmetric ligands were obtained cleanly from this room temperature procedure, with no evidence for pyrazolyl group exchange leading to symmetrically substituted byproducts which can occur under more forcing conditions.^65^ The corresponding iodo-substituted ligand L^I^ is not included in this study, but has been reported previously.^61,66^

Treatment of hydrated Fe[BF_4_]_2_ or Fe[ClO_4_]_2_ with 2 equiv. L^R^ yields [Fe(L^Me^)_2_]Z_2_ (1Z_2_; Z^−^ = BF_4_^−^ or ClO_4_^−^), [Fe(L^F^)_2_]Z_2_ (2Z_2_), and [Fe(L^Br^)_2_]Z_2_ (3Z_2_) as orange or yellow powders after the usual workup.^62^ All the complex salts can be crystallised by vapour diffusion of diethyl ether antisolvent into their nitromethane or acetonitrile solutions. The crystals are air-stable and are usually well-formed, but always diffract poorly. Variable temperature crystallographic characterisation was ultimately achieved from a single crystal of 1[ClO_4_]_2_ using synchrotron radiation. A low temperature structure refinement was also obtained from a synchrotron dataset of a more weakly diffracting crystal of 3[BF_4_]_2_.

Crystals of 1[ClO_4_]_2_ (monoclinic, space group *P*2_1_, *Z* = 2) are isomorphous with its symmetrically substituted analogues [Fe(bpp)_2_][BF_4_]_2_,^67^ [Fe(bpp^H,F^)_2_]Z_2_ (Scheme 1)^49^ and the low-spin forms of [Fe(bpp^H,Y^)_2_]Z_2_ (Y = Me, Cl or Br).^52,68^ Datasets at four temperatures between 100 and 300 K were collected from the same crystal, to monitor its spin state properties over that temperature range. An initial structure solution and refinement at 100 K showed a complex cation on a general crystallographic site. Fourier peaks adjacent to all four pyrazolyl *C*4 atoms suggested disorder of the two methyl groups around these positions in the molecule. However, when this was included in the refinement, geometric distortions and elongated displacement ellipsoids in the ligand framework implied the presence of additional disorder (Fig. S10†). This was addressed with a whole molecule disorder model.

**Fig. 1:**
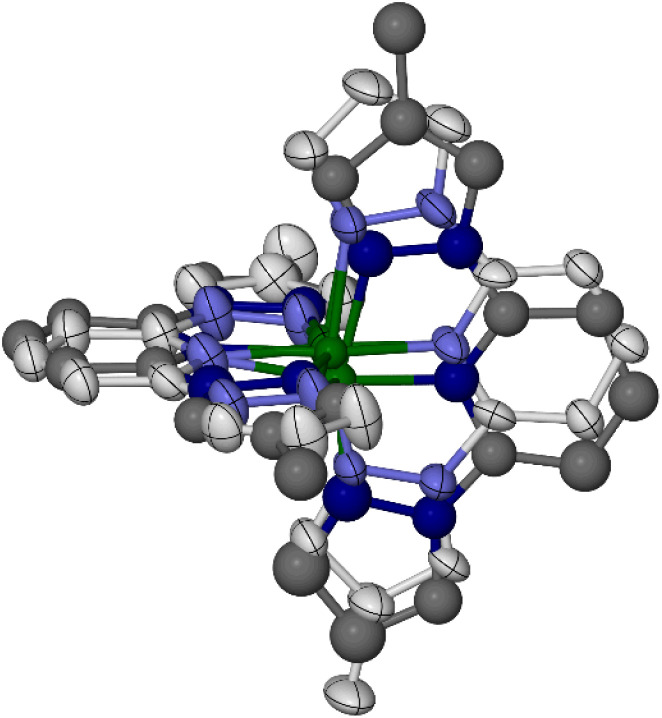
The disordered cation refinement of low-spin 1[ClO_4_]_2_ at 100 K. The major and minor orientations of each ligand are shown with pale and dark coloration, respectively. Three partial Fe atom sites (out of a possible four) are also included in the disorder model. Colour code: C, white or dark grey; Fe, green; N, pale or dark blue.

Two orientations were modelled for each ligand (Fig. 1). Equivalent C–C and C–N bond lengths in each disorder site were constrained to be the same, but no thermal parameter restraints were applied to the model. Different occupancies were refined for the disorder sites of the two L^Me^ ligands, which are not constrained to be equally occupied in the monoclinic lattice site symmetry. One ligand was refined over two orientations with a 0.60 : 0.40 occupancy ratio, while for the other ligand the occupancies were 0.80 : 0.20. This implies the cation is disordered over four positions comprising all possible combinations of each pair of ligand orientations, with occupancies of 0.48, 0.32, 0.12 and 0.08. Attempts to refine four iron atom disorder sites corresponding to these orientations, with reasonable Fe–N bond lengths to each partial ligand, were only partly successful. Three iron atom positions were identified in the Fourier map, and refined with occupancies of 0.50, 0.25 and 0.25. Both perchlorate ions in the model are also disordered, which were treated in the usual way.

Datasets from the same crystal at 180, 220 and 300 K were treated similarly. Refinements based on ordered and disordered complex cations were investigated, with the latter deriving from the same cation disorder model constructed at 100 K. The disordered cation models at 100, 180 and 220 K have lower residuals and more reasonable molecular geometries and displacement parameters than the ordered cation refinements. The two models of the 300 K data have similar residuals, however.

While the disordered cation refinements are a better description of the crystal as a whole, the metric parameters in the disordered model are too imprecise to be useful. Consideration of the averaged iron coordination geometry in the ordered cation models demonstrates that the crystal is low-spin at 100 and 180 K; predominantly low-spin at 220 K; and high-spin at 300 K (Table 1). Hence, the crystal has undergone spin-crossover on warming between 220 and 300 K.

**Table 1:** Selected metric parameters from the crystallographically ordered cation refinements of 1[ClO_4_]_2_.^a^ Full tables of bond lengths and angles are in the ESI

*T*/K	300	220	180	100
Fe–N{pyridyl}/Å	2.112(6), 2.118(6)	1.906(6), 1.911(5)	1.899(5), 1.900(6)	1.886(7), 1.896(6)
Fe–N{pyrazolyl}/Å	2.173(8)–2.208(9)	1.961(7)–2.002(8)	1.964(7)–2.011(10)	1.954(8)–1.993(9)
*V*_Oh_/Å^3^	12.32(3)	9.71(2)	9.66(2)	9.61(3)
*Σ*/deg	152.9(10)	89.0(10)	85.6(10)	84.8(11)
*Θ*/deg	502	293	282	279
*ϕ*/deg	177.3(4)	178.1(3)	178.4(3)	178.4(4)
*θ*/deg	89.99(7)	89.50(7)	89.39(7)	89.28(7)

a *V*_Oh_ is the volume of the octahedron defined by the FeN_6_ coordination sphere.^69^*Σ* is a general measure of the deviation of a metal ion from an ideal octahedral geometry, while *Θ* more specifically indicates its distortion towards a trigonal prismatic structure.^69,70^*ϕ* is the *trans*-N{pyridyl}–Fe–N{pyridyl} bond angle, while *θ* is the dihedral angle between the least squares planes of the two tridentate ligands.^35,67^ More detailed definitions and discussions of these parameters are in the cited references, and in the ESI to this article.

Crystals of 3[BF_4_]_2_ (monoclinic, *P*2_1_, *Z* = 2) are isomorphous with 1[ClO_4_]_2_ but diffract more weakly, and a full refinement was only achieved from synchrotron data at 100 K. The Br substituents are similarly disordered around the complex, but this appears to have little impact on the rest of the molecule (Fig. S12†); attempts to produce a cation disorder model for 3[BF_4_]_2_ were less satisfactory and did not improve the refinement residuals. The refined occupancies of the partial Br atoms yield a 0.63 : 0.25 : 0.09 : 0.03 distribution for the four cation orientations in this crystal. The complex is low-spin at 100 K.

**Fig. 2:**
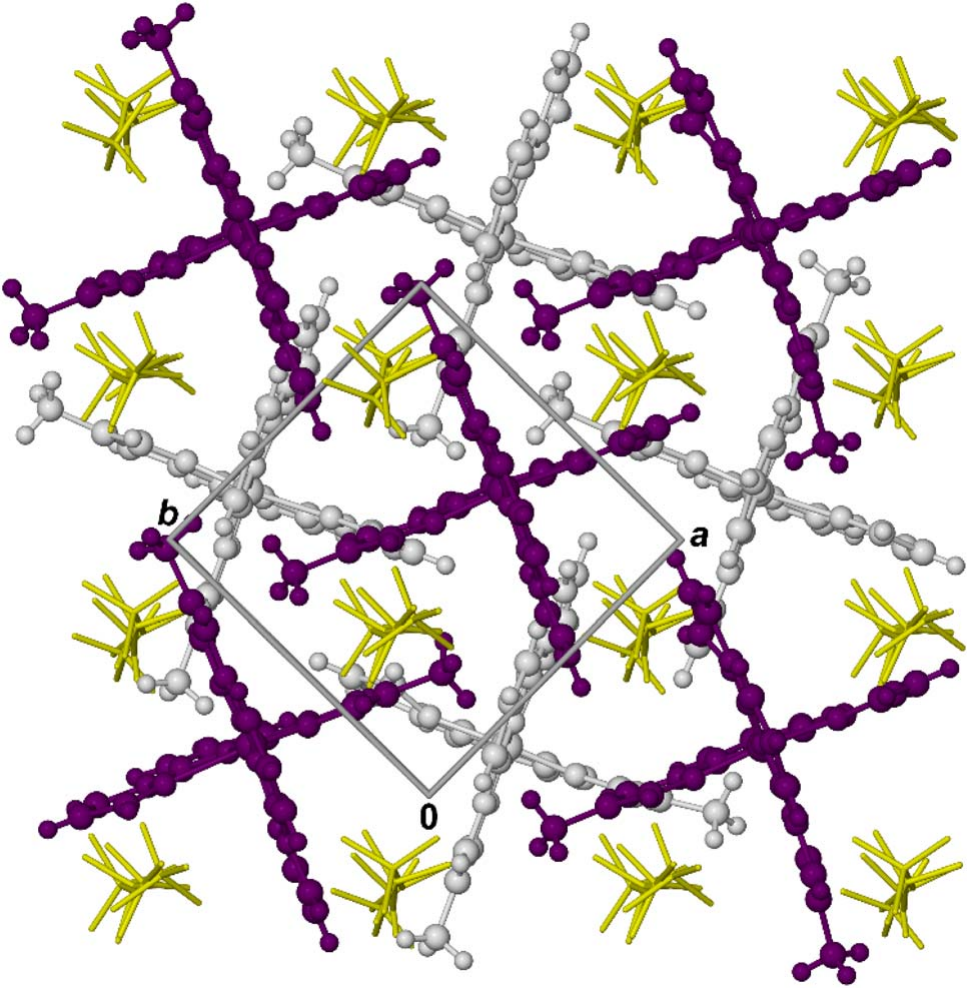
Packing diagram for 1[ClO_4_]_2_ at 100 K viewed parallel to the [001] vector, with a random distribution of cation orientations. Alternate cation layers in the lattice are coloured white and purple, while the ClO_4_^−^ ions (yellow) are de-emphasised for clarity.

Both compounds crystallise in a version of the ‘terpyridine embrace’ structure type^51^ which is also adopted by their isomorphous, symmetrically substituted [Fe(bpp^H,Y^)_2_]Z_2_ counterparts (Scheme 1).^49,52^ The complex cations associate into 2D layers in the (001) plane, through interdigitation of their distal pyrazolyl groups (Fig. 2). The cations are tightly packed within these layers, *via* intermolecular face-to-face π⋯π and edge-to-face C–Y⋯π (Y = H or halogen; Scheme 1) contacts. Neighboring cation layers in the lattice are not in direct van der Waals contact, however, being separated by the counter-anions.

Each molecule in the cation layers has approximate, non-crystallographic 4̄ site symmetry. That is consistent with the *D*_2d_ symmetry of symmetrically substituted [Fe(bpp^X,Y^)_2_]^2+^ molecules, which can also show terpyridine embrace packing (Scheme 1).^49,52–57^ However the unsymmetric ligand substitution pattern in [Fe(L^R^)_2_]^2+^ lowers the molecular point symmetry to *C*_1_, but without changing the lattice site symmetry or the shape of the unit cell. There are no intermolecular steric clashes between nearest neighbour molecules adopting different combinations of disorder sites. Hence, the lattices in 1[ClO_4_]_2_ and 3[BF_4_]_2_ should contain a random distribution of molecular orientations, in the proportions described above (Fig. 2).

The different cation disorder ratios in the two crystals imply that disorder might vary between samples of the same material, or in different crystallites from the same sample. To minimise that uncertainty, all physical characterisation was performed on (poly)crystalline samples grown from nitromethane/diethyl ether using the same vapour diffusion crystallisation method.

Room temperature X-ray powder diffraction patterns from all the complex salts were weak with significant peak broadening, showing the compounds are poorly crystalline. Simulation of these powder patterns are consistent with the single crystal data, and showed all the compounds are isomorphous and phase-pure (Fig. S13 and Table S5†). Single crystal unit cell data from two other compounds at low temperature showed they also retain the same phase in the low-spin state (Table S2†).

**Fig. 3:**
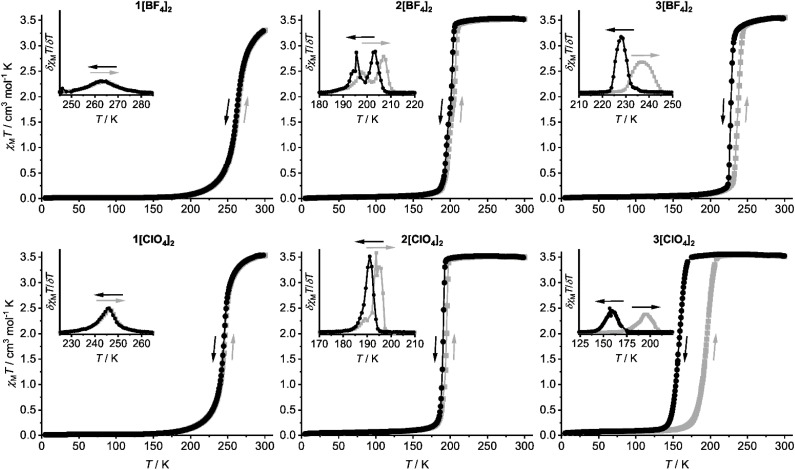
Magnetic susceptibility data for the compounds in this work, measured in cooling (black) and warming (grey) modes at a scan rate of 2 K min^−1^. Datapoints are connected by spline curves for clarity. The insets show the first derivative of each *χ*_M_*T vs. T* plot. These insets are all plotted to the same horizontal and vertical scales, except the 3[ClO_4_]_2_ inset which spans a larger temperature range reflecting its wider thermal hysteresis.

Variable temperature magnetic susceptibility data show each compound is high-spin at room temperature, but exhibits thermal SCO to their low-spin form on cooling (Fig. 3 and Table 2). The form of this SCO differs strongly between the samples, however. SCO for 1[BF_4_]_2_ (*T*_1/2_ = 263 K) and 1[ClO_4_]_2_ (*T*_1/2_ = 245 K) occurs gradually with temperature, continuously and without hysteresis. That is consistent with the crystallographic data from the perchlorate salt (Table 1). In contrast, 2[BF_4_]_2_ (*T*_1/2_ = 201 K) and 2[ClO_4_]_2_ (*T*_1/2_ = 193 K) undergo abrupt spin-transitions with little or no hysteresis that appear to occur in one step (Fig. 3). First derivatives of their *χ*_M_*T vs. T* curves reveal hidden structure a 10–15 K temperature range. The steps are distributed irregularly across the transition, and occur differently in cooling and warming modes.

**Table 2:** Spin-transition parameters from the magnetic susceptibility data of the complexes in this work (Fig. 3)

	*T*_1/2_↓/K	*T*_1/2_↑/K	Form
1[BF_4_]_2_	262	263	Gradual
1[ClO_4_]_2_	245	245	Gradual
2[BF_4_]_2_	200	202	Abrupt^a^
2[ClO_4_]_2_	190	194	Abrupt^a^
3[BF_4_]_2_	228	237	Abrupt
3[ClO_4_]_2_	158	194	Abrupt

a These transitions contain hidden structure, which is revealed by the first derivative plots of the magnetic data (Fig. 3).

Solid 3Z_2_ also exhibit abrupt spin-transitions below room temperature, but now with significant thermal hysteresis (Table 2). The hysteresis in 3[BF_4_]_2_ (Δ*T*_1/2_ = 9 K) is asymmetric, with a more gradual warming branch whose first derivative again shows evidence of structure. The hysteresis loop in 3[ClO_4_]_2_ is wider (Δ*T*_1/2_ = 36 K), but more typically symmetrical.

The SCO transitions were also monitored by differential scanning calorimetry (DSC), which was measured on a warming temperature ramp at a scan rate of 5 K min^−1^. All the compounds were investigated except 3[ClO_4_]_2_, whose SCO occurs below 183 K, the low-temperature limit of our calorimeter (Table 2). Each DSC trace shows an exothermic anomaly at a temperature corresponding to the low → high-spin transition in the magnetic data (Tables 2 and 3). The exotherms for 1[BF_4_]_2_ and 1[ClO_4_]_2_ were broad and symmetrical, as expected for gradual and continuous SCO. The peaks for the other, more cooperative compounds were sharper and structured, as expected from the magnetic data. The anomalies for 2[BF_4_]_2_ and 3[BF_4_]_2_ are split into two unequal components, while a broader low-temperature shoulder is also visible on the peak for 2[ClO_4_]_2_.

**Table 3:** Low → high-spin transition parameters from the DSC measurements (Fig. 4, S14 and S15). Data for 3[ClO_4_]_2_ were not measured, because its SCO occurs below the low-temperature limit of our calorimeter (183 K)

	T_1/2_↑/K	Δ*H*↑/kJ mol^−1^	Δ*S*↑/J mol^−1^ K^−1^
1[BF_4_]_2_	263.5	11.2	42.5
1[ClO_4_]_2_	246.4	11.1	44.9
2[BF_4_]_2_	203.5, 209.6	3.3, 11.5	16.4, 54.6
2[ClO_4_]_2_	194,^a^ 196.6	1.4, 10.3^b^	7.4, 52.2^b^
3[BF_4_]_2_	233.8, 240.0	3.8, 11.1	16.1, 46.5

a Broad low-temperature shoulder on the main peak.

b These thermodynamic data may be underestimated, because the peaks are close to the low-temperature limit of the measurement.

**Fig. 4:**
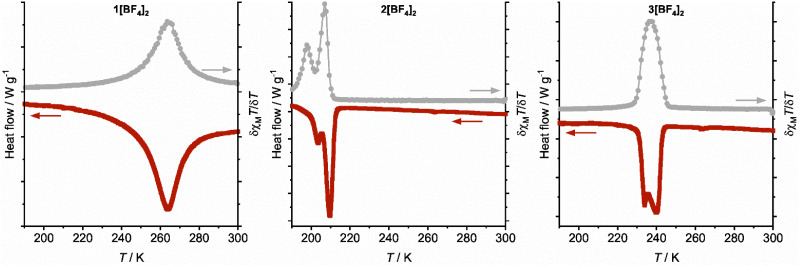
Comparison of the DSC data for the BF_4_^−^ salts in this work (red), with the first derivative of the magnetic data from Fig. 3 (grey). All data were measured on a warming temperature ramp (the arrows in this figure indicate the relevant *y* axis scale for each plot). The equivalent plots for the ClO_4_^−^ salts are shown in Fig. S15.† The DSC data were measured on a 5 K min^−1^ temperature ramp.

The form and the temperature of the DSC anomalies for all the samples are in excellent agreement with the first derivative plots of the corresponding magnetic data (Fig. 4 and S15†). The only small discrepancy is for 2[BF_4_]_2_, whose two DSC anomalies occur at 3–5 K higher temperature than predicted by the magnetic data. That could reflect the different thermal scan rates in the measurements^71^ or different crystallite sizes in the samples used, which were recrystallised for the DSC study.^72–80^ Both these factors are known to influence the temperature of cooperative SCO transitions.

**Fig. 5:**
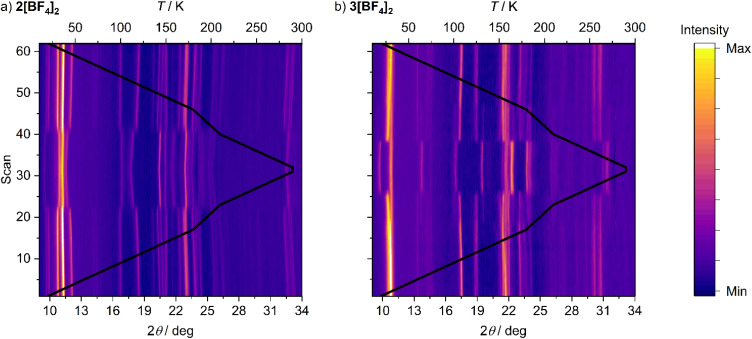
Variable temperature X-ray powder diffraction data for 2[BF_4_]_2_ (left) and 3[BF_4_]_2_ (right), showing the shifts of the Bragg peak positions associated with SCO. Data were collected on a 20–290–20 K temperature ramp at 10 K intervals, with additional data every 5 K between 180–210 K; the black line indicates the temperature of each scan, scaled against the top axis. Representative variable temperature powder patterns for these and other compounds are in Fig. S16–S20.†

A possible explanation for the discontinuous spin-transitions in 2[BF_4_]_2_, 2[ClO_4_]_2_ and 3[BF_4_]_2_ (Fig. 3 and 4) could be the involvement of a crystallographic phase change in the SCO process.^81,82^ This was probed by variable temperature powder diffraction on those three compounds, and on the crystallographically characterised complex 1[ClO_4_]_2_. Each sample underwent SCO at the temperatures predicted by their magnetic data, without a crystallographic phase change (Fig. 5 and S16–S20†). The low-temperature data from 1[ClO_4_]_2_ and 3[BF_4_]_2_ agree well with simulations from their single crystal structures. At temperatures close to *T*_1/2_2[BF_4_]_2_, 2[ClO_4_]_2_ and 3[BF_4_]_2_ are a mixture of their high-spin and low-spin forms. In contrast the gradual SCO material 1[ClO_4_]_2_ displays just one set of Bragg peaks at all temperatures, whose positions evolve continuously around *T*_1/2_ as the transition proceeds. There is no evidence for a re-entrant intermediate crystal phase, that could explain the structured transitions in Fig. 3, in any of these data.^83–93^

The main transition between the high- and low-spin phases in 3[BF_4_]_2_ occurs between 230–220 K on cooling and between 230–250 K on warming, which reproduces the hysteresis in its magnetic data (Fig. 3). However, a minor fraction of high-spin material is visible between 170–210 K on the heating and cooling cycles, when the sample is predominantly low-spin (Fig. 5b). That high-spin residue disappears below 170 K on cooling, and reappears around 190 K on warming. Below those temperatures, the powder patterns agree well with a simulation based on its low-spin single crystal structure.

That implies a small fraction of 3[BF_4_]_2_ undergoes SCO at lower temperature, or more gradually, than the rest of the sample. Since the sample was phase-pure, we interpret this as additional structure in its spin-transition. While a lower temperature SCO component isn't unambiguously visible in the magnetic or DSC data, the low-temperature limit of our DSC measurements could have prevented its observation by that technique. There is no evidence for a high-spin residue below *T*_1/2_ in the data for 1[ClO_4_]_2_, 2[BF_4_]_2_ or 2[ClO_4_]_2_.

## Discussion

Several [Fe(bpp^X,Y^)_2_]Z_2_ derivatives (Scheme 1) with symmetric patterns of ligand substituents are either isomorphous with 1Z_2_-3Z_2_, or adopt closely related forms of terpyridine embrace crystal packing.^47,49,52–57,67,68^ These compounds usually exhibit abrupt thermal spin-transitions, with a narrow thermal hysteresis.^47,49,52,53,67,68^ In that respect, the more varied SCO properties shown by 1Z_2_-3Z_2_ are unexpected. For example, gradual SCO as shown by 1[BF_4_]_2_ and 1[ClO_4_]_2_ has only been observed once before in iron(ii) complexes with this structure type, namely [Fe(bpp^CH2OH,H^)_2_]Z_2_ (Z^−^ = BF_4_^−^ or ClO_4_^−^; Scheme 1).^55^ Disorder of the hydroxymethyl ligand substituents suggests a less dense crystal packing in those literature compounds, which should contribute to their less cooperative SCO.

Tetra-substituted [Fe(bpp^H,Me^)_2_][ClO_4_]_2_ and [Fe(bpp^H,Br^)_2_][BF_4_]_2_ (Scheme 1) also form terpyridine embrace crystals, but exhibit typically abrupt thermal spin-transitions.^52,68^ In comparison, each cation in 1[ClO_4_]_2_ and 3[BF_4_]_2_ has only half as many methyl or bromo substituents, to mediate intermolecular contacts between molecules in their cation layers (Fig. 2). The molecular disorder in those crystals could also reflect less dense molecular packing within their cation layers, which might contribute to the less cooperative SCO in 1[BF_4_]_2_ and 1[ClO_4_]_2_.

Their cation packing can be quantified *via* the area of the 2D cation layers as expressed by *ab*, the product of the *a* and *b* unit cell dimensions (Fig. 2).^94,95^ Adding pairs of methyl substituents to the high-spin [Fe(bpp^H,Y^)_2_]Z_2_ scaffold (Scheme 1) expands their cation layers by a consistent amount, equivalent to Δ*ab* ≈ 10 Å^2^ (Table 4). Similarly, sequentially adding each pair of bromo substituents increases to Δ*ab* by a slightly larger amount, of *ca* 11.5 Å^2^. The analysis could not be performed for the low-spin states, because data are not available for all the compounds at consistent temperatures. However, these data confirm high-spin 1[ClO_4_]_2_ and 3[BF_4_]_2_ have the expected crystal packing density, based on their chemical compositions. So the existence of molecular disorder in those crystals, and their different SCO properties, do not reflect a less dense crystal packing.

**Table 4:** Crystallographic area of the cation layers in high-spin [Fe(bpp^X,Y^)_2_]Z_2_ crystals (Scheme 1), as a function of the number of ‘Y’ substituents

	*T*/K	No. of Y groups	*ab*/Å^2^	Ref.
**Y = Me**				
[Fe(bpp)_2_][BF_4_]_2_	290	0	72.2644(6)	67
1[ClO_4_]_2_	300	2	82.5944(12)	This work
[Fe(bpp^H,Me^)_2_][ClO_4_]_2_	250	4	91.901(2)^a^	68

**Y = Br**				
[Fe(bpp)_2_][BF_4_]_2_	290	0	72.2644(6)	67
3[BF_4_]_2_	300	2	84.0614(9)	This work
[Fe(bpp^H,Br^)_2_][BF_4_]_2_	300	4	95.2869(2)	52

a Data from other [M(bpp)_2_]Z_2_ (M^2+^ = a transition ion) crystals with a terpyridine embrace lattice suggest *ab* should be *ca* 0.4 Å^2^ larger at 300 K than this value measured at 250 K.^94,95^

C–H⋯π interactions are one of the weakest types of hydrogen bond.^96,97^ The energies of C–Y⋯π contacts (Y = halogen) are similarly small, and steric considerations appear to be most important when comparing interactions of this type.^98,99^ The enthalpies of different C–Y⋯π interactions in solution follow the trend Y = CH_3_ > F > Cl ≈ Br > I.^98^ Since the SCO cooperativity in 1Z_2_-3Z_2_ follows the opposite trend in Y (Fig. 3), this does not correlate with the strength of these intermolecular interactions in their crystal lattices.

Published structures of symmetrically substituted [Fe(bpp^H,Y^)_2_]Z_2_ show that Me, F, Cl and Br ‘Y’ substituents weaken the π⋯π contacts within the terpyridine embrace layers by a largely consistent amount (Table 5). Hence, while the crystal packing in 1Z_2_-3Z_2_ cannot be directly compared, there is no evidence that the local environment about each molecule in 1Z_2_-3Z_2_ should strongly depend on the identity of their ligand substituents.

**Table 5:** Intermolecular π⋯π and C–Y⋯π distances within the cation layers of [Fe(bpp^H,Y^)_2_]Z_2_ terpyridine embrace crystals in their high-spin state (Scheme 1)

	Y	*T*/K	π⋯π/Å	Y⋯π/Å	Ref.
[Fe(bpp)_2_][BF_4_]_2_^a^	H	290	3.353(15), 3.565(11)	2.73–3.12	67
[Fe(bpp^H,Me^)_2_][ClO_4_]_2_^b^	Me	250	3.68	2.90^c^	68
[Fe(bpp^H,F^)_2_][BF_4_]_2_^b^	F	300	3.68(3)	2.965(9)	49
[Fe(bpp^H,F^)_2_][ClO_4_]_2_^a^	F	250	3.69(3), 3.84(3)	2.91(1)–3.06(1)	49
[Fe(bpp^H,Cl^)_2_][BF_4_]_2_^b^	Cl	300	3.642(12)	3.181(3)	52
[Fe(bpp^H,Br^)_2_][BF_4_]_2_^b^	Br	300	3.665(19)	3.228(4)	52

a The complex molecule in this crystal has no internal symmetry, and participates in two unique π⋯π and four unique C–Y⋯π contacts.

b The complex molecule in this crystal has crystallographic 4̄ symmetry, giving just one unique intermolecular interaction of each type.

c Intermolecular H{Me}⋯C{pyrazolyl} distance.

Another structural factor that could be relevant, is the degree of cation disorder in each 1Z_2_-3Z_2_ crystal. This disorder is greater in 1[ClO_4_]_2_ than in 3[BF_4_]_2_, based on their low temperature structure refinements. That implies the complex molecules in 1[ClO_4_]_2_ occupy a more heterogeneous distribution of lattice sites, which would be consistent with less cooperative SCO.^77,100^ Structural data from the other compounds would be required to show if this is a general trend in these materials, however.

Terpyridine embrace crystals exhibiting discontinuous (stepped) spin-transitions are also known in the [Fe(bpp^X,Y^)_2_]Z_2_ system.^54,56^ However, their discontinuities occur identically on both cooling and warming at around 50% conversion, when the sample has a 1 : 1 high : low-spin state population; that is the most common scenario in materials showing discontinuous SCO.^81,82^ Such discontinuities usually reflect a crystallographic phase change around the midpoint of the transition,^83–93^ or the existence of two independent switching centers in the material.^101–106^

In contrast, the irregular and unsymmetric pattern of discontinuities in the spin-transitions of 2[BF_4_]_2_, 2[ClO_4_]_2_ and 3[BF_4_]_2_ is highly unusual for phase-pure SCO materials (Fig. 3 and 4). The discontinuities in this study could originate from populations of molecules in different local environments, undergoing abrupt SCO at sightly different temperatures. Supporting that, intermolecular intralayer steric contacts are known to induce discontinuous SCO in two other terpyridine embrace crystals.^58,107^ However, it is surprising that the same cation disorder could lead to such widely variable SCO properties in 1Z_2_-3Z_2_. This includes, in 3[ClO_4_]_2_, the widest thermal hysteresis for a [Fe(bpp)_2_]^2+^ derivative in a terpyridine embrace lattice.^47,49,52–57,67,68,108^

## Conclusions

Unsymmetrically substituted [Fe(L^R^)_2_]Z_2_ (R = Me, 1Z_2_; R = F, 2Z_2_; R = Br, 3Z_2_; Z^−^ = BF_4_^−^ or ClO_4_^−^) are isomorphous, and adopt the same mode of crystal packing as their symmetrically substituted analogues [Fe(bpp^H,Y^)_2_]Z_2_ (Scheme 1).^62^ Structural data from 1[ClO_4_]_2_ and 3[BF_4_]_2_ show non-statistical disorder of the L^R^ ligand substituents, which leads to resolvable whole-molecule disorder in the crystal of 1[ClO_4_]_2_. Solid 1Z_2_-3Z_2_ are high-spin at room temperature but show an unexpected variety of SCO behaviors on cooling, including gradual SCO (1Z_2_); abrupt spin-transitions with unusual, irregular discontinuities (2Z_2_ and 3[BF_4_]_2_); and more conventional hysteretic spin-transitions with hysteresis widths up to 36 K (3[ClO_4_]_2_).

The available data show SCO in 1Z_2_-3Z_2_ does not involve a crystallographic phase change, and that the compounds are isomorphous with each other in both spin states. Rather, there is some evidence that their different spin state properties relate to the degree of cation disorder in each material. Firstly, compounds with more cation disorder (like 1[ClO_4_]_2_) may show less cooperative SCO, and *vice versa*. Secondly, the unusual discontinuous transitions in 2[BF_4_]_2_, 2[ClO_4_]_2_ and 3[BF_4_]_2_ could reflect local structural heterogeneities within those disordered, cooperative SCO materials.

This cation disorder might depend on the method of sample preparation and could be manipulated, for example by rapid precipitation *vs.* slow crystallisation. While that could be difficult to quantify, it would imply SCO in these materials could be especially sensitive to sample history.^72–80^ That merits further investigation.

## Data availability

Data supporting this study are available in the ESI,† or at http://doi.org/10.5518/1625.

## Author contributions

The synthesis and analytical characterisation of the compounds was performed by HES and RLW. HBV and NS undertook the magnetic measurements, using SQUID magnetometer time provided by OC. CMP collected and processed the single-crystal diffraction data at the Diamond synchrotron, and MAH performed the structure refinements and analyses. DLB measured the DSC data and assisted with its analysis, while DS and ICB did the variable temperature powder diffraction investigation. MAH conceived and supervised the study, and prepared the publication. All authors have approved the final version of the manuscript.

## Conflicts of interest

There are no conflicts to declare.

## Supplementary Material

SC-OLF-D5SC00090D-s001

SC-OLF-D5SC00090D-s002
