# Supplementary material for: The impact of whole-molecule disorder on spin-crossover in a family of isomorphous molecular crystals
Source: Chem Sci. 2025 Apr 10;16(21):9203–12. doi: 10.1039/d5sc00090d (PMC12023404; doi:10.1039/d5sc00090d)
Supplement: SC-016-D5SC00090D-s001 [file SC-016-D5SC00090D-s001.pdf]

Supplementary Information (SI) for Chemical Science.
This journal is © The Royal Society of Chemistry 2025

Supporting Information

The Impact of Whole-Molecule Disorder on Spin-Crossover in a Family of Isomorphous Molecular Crystals

Holly E. Sephton,[a,+] Rhiannon L. Watson,[a,+] Namrah Shahid,[a,b] Hari Babu Vasili,[c] Daniel L. Baker,[c] Dipankar Saha,[d] Izar Capel Berdiell,[e] Christopher M. Pask,[a] Oscar Cespedes[c] and Malcolm A. Halcrow*[a]

[a]*School of Chemistry, University of Leeds, Woodhouse Lane, Leeds LS2 9JT, United Kingdom.*
E-mail: m.a.halcrow@leeds.ac.uk

[b]*Current address: Department of Chemistry, University of Durham, Durham DH1 3LE, United Kingdom.*

[c]*School of Physics and Astronomy, W. H. Bragg Building, University of Leeds, Leeds LS2 9JT, United Kingdom.*

[d]*Department of Chemistry, Faculty of Mathematics and Natural Sciences, University of Oslo, 0371 Oslo, Norway.*

[e]*Center for Material Science and Nanomaterials (SMN), University of Oslo, 0371 Oslo, Norway.*

[+]These authors contributed equally to this study.

		Page
**Experimental − Synthesis and Characterisation**		S3
**Experimental – Crystal Structure Determinations**		S4
**Experimental – Other Measurements**		S5
**Table S1**	Experimental data for the crystal structure determinations of **[1ClO$_4$]$_2$**.	S6
**Table S2**	Experimental data for the other crystal structure determinations.	S7
**Figure S1**	^{1}H and ^{13}C NMR spectra of L^{Me}.	S8
**Figure S2**	^{1}H, ^{13}C and ^{19}F NMR spectra of L^F.	S9
**Figure S3**	^{1}H and ^{13}C NMR spectra of L^{Cl}.	S11
**Figure S4**	^{1}H and ^{13}C NMR spectra of L^{Br}.	S12
**Figure S5**	Paramagnetic ^{1}H NMR spectrum of **1[BF$_4$]$_2$**.	S13
**Figure S6**	Paramagnetic ^{1}H NMR spectrum of **2[BF$_4$]$_2$**.	S13
**Figure S7**	Paramagnetic ^{1}H NMR spectrum of **3[BF$_4$]$_2$**.	S14
**Figure S8**	Paramagnetic ^{1}H NMR spectrum of [Fe(L^{Cl})$_2$][BF$_4$]$_2$	S14
**Definitions of the structural parameters discussed in the paper**		S15
**Scheme S1**	Angles used in the definitions of the coordination distortion parameters Σ and Θ.	S15
**Scheme S2**	Definition of the Jahn-Teller distortion parameters θ and ϕ.	S15
**Figure S9**	The asymmetric unit of the ordered cation refinement of **1[ClO$_4$]$_2$** at 100 K.	S16
**Figure S10**	Comparison of the ordered and disordered cation refinements of **1[ClO$_4$]$_2$** at each temperature.	S16
**Table S3**	Selected bond lengths, angles and other metric parameters for the ordered cation refinements of **1[ClO$_4$]$_2$**.	S18
**Figure S11**	Comparison of the spin-transition in **1[ClO$_4$]$_2$** as measured from magnetic susceptibility data and its ordered cation crystallographic refinements.	S18
**Figure S12**	The asymmetric unit of **3[BF$_4$]$_2$** at 100 K.	S19
**Table S4**	Selected bond lengths, angles and other metric parameters for **3[BF$_4$]$_2$**.	S19
**Figure S13**	Room temperature X-ray powder diffraction data for the complexes **1Z$_2$-3Z$_2$**.	S20
**Table S5**	Unit cell parameters refined from the powder diffraction data.	S21
**Figure S14**	DSC data for the complexes **1Z$_2$-3Z$_2$**, measured in warming mode.	S22
**Figure S15**	Comparison of the DSC and magnetic data for **1[ClO$_4$]$_2$** and **2[ClO$_4$]$_2$**.	S23
**Figure S16**	Full variable temperature powder diffraction data for **1[ClO$_4$]$_2$**.	S24
**Figure S17**	Representative variable temperature powder patterns for **1[ClO$_4$]$_2$**.	S25
**Figure S18**	Representative variable temperature powder patterns for **2[BF$_4$]$_2$**.	S26
**Figure S19**	Variable temperature powder patterns for **2[ClO$_4$]$_2$**.	S27
**Figure S20**	Representative variable temperature powder patterns for **3[BF$_4$]$_2$**.	S28
**Figure S21**	Room temperature X-ray powder diffraction data for [Fe(L^{Cl})$_2$][BF$_4$]$_2$.	S29
**Figure S22**	Magnetic susceptibility data for [Fe(L^{Cl})$_2$][BF$_4$]$_2$.	S29
**References**		S30

Experimental

The ligand precursor 2-(pyrazol-1-yl)-6-fluoropyridine was prepared by the literature procedure.[1,2] Other reagents were purchased commercially and used as supplied.

Synthesis of 2-(pyrazol-1-yl)-6-(4-methylpyrazol-1-yl)pyridine (L^{Me}). 4-Methyl-1*H*-pyrazole (0.70 g, 8.54 mmol) was dissolved in a 3:1 *N*,*N*-dimethylformamide:tetrahydrofuran solvent mixture (12 cm^3) under an N2 atmosphere. NaH (60% dispersion in mineral oil; 0.35 g, 8.75 mmol) was slowly added to the reaction mixture which was then left to stir for 10 minutes until the evolution of hydrogen gas had ceased. 2-(Pyrazol-1-yl)-6-fluoropyridine (1.4 g, 8.35 mmol) was then added, producing a cloudy pale-yellow solution. The mixture was left to stir for 18 h at room temperature. Water (100 cm^3) was then added, affording a white precipitate which was collected by filtration, washed with water and pentane then dried *in vacuo*. Yield 1.0 g, 55 %. ESI-MS *m/z* 226.1085 (calcd for $[HL^{Me}]^+$ *m/z* 226.1087), 248.1013 (calcd for $[NaL^{Me}]^+$ *m/z* 248.0912), 473.1926 (calcd for $[Na(L^1)_2]^+$ *m/z* 473.1927). ^{1}H NMR ($CDCl_3$) δ 2.13 (s, 3H, CH_3), 6.43 (dd, 1H, Pz H^4), 7.52 (s, 1H, Pz H^3), 7.70 (pseudo-t, 1H, Pz H^3), 7.74, 7.76 (both d, 1H, Py H^3 and H^5), 7.84 (pseudo-t, 1H, Py H^4), 8.27 (d, 1H, Pz H^5), 8.52 (dd, 1H, Pz H^5) ppm. ^{13}C NMR ($CDCl_3$) δ 9.1(CH_3), 107.9, 109.0, 109.0 (Py C^3 and C^5 + Pz C^4H), 118.7 (Pz C^4Me), 125.6, 127.1 (2x Pz C^5), 141.3, 142.3, 143.3 (Py C^4 + 2x Pz C^3), 150.0 (Py C^2 and C^6) ppm.

Synthesis of 2-(pyrazol-1-yl)-6-(4-fluoropyrazol-1-yl)pyridine (L^F). Method as for L^{Me}, using 4-fluoro-1*H*-pyrazole (0.72 g, 8.54 mmol). The full reaction mixture was stirred at room temperature for 48 hrs, then worked up as described above. Yield 1.2 g, 63 %. ESI-MS *m/z* 230.0822 (calcd for $[HL^F]^+$ *m/z* 230.0836), 252.0661 (calcd for $[NaL^F]^+$ *m/z* 252.0678). ^{1}H NMR ($CDCl_3$) δ 6.45 (dd, 1H, Pz H^4), 7.56 (d, 1H, Pz H^3), 7.71 (d, 1H, Pz H^3), 7.77, 7.83 (both d, 1H, Py H^3 and H^5), 7.86 (pseudo-t, 1H, Py H^4), 8.35 (dd, 1H, Pz H^5), 8.48 (dd, 1H, Pz H^5) ppm. ^{13}C NMR ($CDCl_3$) δ 108.2, 108.6, 109.6 (all s, Py C^3 and C^5 + Pz C^4H), 112.8 (d, Pz C^4F), 127.0 (s, Pz C^5), 130.4 (d, Pz C^5), 141.4, 142.5 (both s, Py C^4 + Pz C^3), 149.9 (d, Pz C^3), 149.9, 152.4 (both s, Py C^2 and C^6) ppm. ^{19}F NMR ($CDCl_3$) δ –173.9 ppm.

Synthesis of 2-(pyrazol-1-yl)-6-(4-chloropyrazol-1-yl)pyridine (L^{Cl}). Method as for L^{Me}, using 4-chloro-1*H*-pyrazole (0.88 g, 8.54 mmol). The product was a white solid. Yield 0.86 g, 42 %. ESI-MS *m/z* 246.0543 (calcd for $[HL^{Cl}]^+$ *m/z* 246.0541), 268.0358 (calcd for $[NaL^{Cl}]^+$ *m/z* 268.0366). ^{1}H NMR ($CDCl_3$) δ 6.44 (dd, 1H, Pz H^4), 7.60 (s, 1H, Pz H^3), 7.70 (d, 1H, Pz H^3), 7.73, 7.81 (both d, 1H, Py H^3 and H^5), 7.84 (pseudo-t, 1H, Py H^4), 8.47 (m, 2H, 2x Pz H^5) ppm. ^{13}C NMR ($CDCl_3$) δ 108.2, 108.9, 109.9 (Py C^3 and C^5 + Pz C^4H), 113.2 (Pz C^4Cl), 124.9, 127.0 (2x Pz C^5), 140.8, 141.6, 142.6 (Py C^4 + 2x Pz C^3), 149.4, 150.1 (Py C^2 and C^6) ppm.

Synthesis of 2-(pyrazol-1-yl)-6-(4-bromopyrazol-1-yl)pyridine (L^{Br}). Method as for L^{Me}, using 4-bromo-1*H*-pyrazole (1.2 g, 8.54 mmol). The product was a white solid. Yield 1.4 g, 56 %. ESI-MS *m/z* 311.9841 (calcd for $[NaL^{Br}]^+$ *m/z* 311.9861). ^{1}H NMR ($CDCl_3$) δ 6.54 (dd, 1H, Pz H^4), 7.73 (s, 1H, Pz H^3), 7.80 (d, 1H, Pz H^3), 7.86, 7.92 (both d, 1H, Py H^3 and H^5), 7.98 (pseudo-t, 1H, Py H^4), 8.59 (m, 2H, 2x Pz H^5) ppm. ^{13}C NMR ($CDCl_3$) δ 96.7 (Pz C^4Br), 108.2, 108.9, 110.0 (Py C^3 and C^5 + Pz C^4H), 127.1, 127.2 (2x Pz C^5), 141.6, 142.6, 142.8 (Py C^4, 2x Pz C^3). The compound was too insoluble for the quaternary Py C^2 and C^6 environments to be visible by ^{13}C NMR.

Synthesis of the complexes. The following method, described for $[Fe(L^{Me})_2][BF_4]_2$, was followed for all the complexes. A solution of L^{Me} (0.10 g, 0.44 mmol) and $Fe[BF_4]_2\cdot6H_2O$ (0.075 g, 0.22 mmol) in MeCN (10 cm^3) was stirred at room temperature until all the solid had dissolved. The solution was concentrated to *ca* half its original volume, then filtered. Addition of diethyl ether (15 cm^3) to the filtered solution afforded the product as an orange powder. Yield 0.11 g, 73 %.

The same procedure, using equivalent quantities of the appropriate ligand or iron salt, afforded the other complexes in this work. Yields ranged from 62-85 %. All physical characterisation was done on (poly)-crystalline samples of the complexes, which were obtained from nitromethane solution by slow diffusion of diethyl ether antisolvent vapour over a period of 2-3 days.

For $[Fe(L^{Me})_2][BF_4]_2$ (**1[BF$_4$]$_2$**). Orange solid. Found C, 42.3; H, 3.36; N, 20.4 %. Calcd for $C_{24}H_{22}B_2F_8FeN_{10}$ C, 42.4; H, 3.26, N, 20.6 %. ^{1}H NMR (CD_3CN) δ 3.2 (2H), 5.9 (6H), 33.5 (2H), 35.8 (4H), 36.3 (2H), 55.2 (4H), 59.5 (2H);

For $[Fe(L^{Me})_2][ClO_4]_2$ (**1[ClO$_4$]$_2$**). Orange solid. Found C, 40.8; H, 3.11; N 20.2 %. Calcd for $C_{24}H_{22}Cl_2FeN_{10}O_8$ C, 40.9; H, 3.14; N, 19.9 %.

For $[Fe(L^{F})_2][BF_4]_2$ (**2[BF$_4$]$_2$**). Yellow solid. Found C, 38.4; H, 2.55; N, 20.7 %. Calcd for $C_{22}H_{16}B_2F_{10}FeN_{10}$ C, 38.4; H, 2.34; N, 20.4 %. ^{1}H NMR (CD_3CN) δ 1.0 (2H), 29.7 (2H), 40.5 (2H), 52.9 (2H), 60.6 (2H), 62.2 (2H), 64.0 (2H), 71.0 (2H).

For $[Fe(L^{F})_2][ClO_4]_2$ (**2[ClO$_4$]$_2$**). Yellow solid. Found C, 36.7; H, 2.10; N, 19.3 %. Calcd for $C_{22}H_{16}Cl_2F_2FeN_{10}O_8$ C, 37.1; H, 2.26; N, 19.6 %.

For $[Fe(L^{Br})_2][BF_4]_2$ (**3[BF$_4$]$_2$**). Yellow solid. Found C, 32.4; H, 1.80; N, 17.0 %. Calcd for $C_{22}H_{16}B_2Br_2F_8FeN_{10}$ C, 32.6; H, 1.99; N, 17.3 %. ^{1}H NMR (CD_3CN) δ 1.6 (2H), 28.1 (2H), 39.5 (2H), 46.0 (2H), 52.3 (2H), 59.7 (2H), 62.3 (2H), 68.4 (2H).

For $[Fe(L^{Br})_2][ClO_4]_2$ (**3[ClO$_4$]$_2$**). Yellow solid. Found C, 31.5; H, 1.82; N, 16.9 %. Calcd for $C_{22}H_{16}Br_2Cl_2FeN_{10}O_8$ C, 31.7; H, 1.93; N, 16.8 %.

For $[Fe(L^{Cl})_2][BF_4]_2$. Yellow solid. Found C, 36.7; H, 2.12; N, 19.2 %. Calcd for $C_{22}H_{16}B_2Cl_2F_8FeN_{10}$ C, 36.7; H, 2.24; N, 19.4 %. ^{1}H NMR (CD_3CN) δ 1.4 (2H), 28.2 (2H), 39.8 (2H), 47.4 (2H), 54.4 (2H), 60.4 (2H), 63.0 (2H), 69.3 (2H). Selected data for this compound are shown in Figures S21 and S22.

Caution. Although we have experienced no problems when using the perchlorate salts in this study, metal–organic perchlorates are potentially explosive and should be handled with care in small quantities.

Single Crystal Structure Analyses

The single crystals were grown by slow diffusion of diethyl ether vapour into nitromethane solutions of the compounds, as above. All complete X-ray datasets were recorded at station I19 of the Diamond synchrotron (λ = 0.6889 Å); an Agilent SuperNova diffractometer with Cu-K_α (λ = 1.5418 Å) radiation was also used for preliminary crystal screening. Experimental details of the structure determinations in this study are given in Tables S1 and S2. All the structures were solved by direct methods *(SHELXTL*[3]), and developed by full least-squares refinement on F^2 (*SHELXL-2018*[4]). Crystallographic figures were prepared using *XSEED*,[5] and materials for publication were prepared with *Olex2*.[6]

Structure refinements of 1[ClO$_4$]$_2$. Datasets were collected from the same crystal at four temperatures: 300, 220, 180 and 100 K. The crystal retains the same crystal phase across this temperature range, with one formula unit per asymmetric unit. The cation and two anion residues all lie on general crystallographic sites.

The crystal diffracted weakly, with fraction of observed data ranging from 26 % at 300 K to 45 % at 100 K. Despite that limitation, the observed data:parameter ratios in the following refinements lie between 7.0:1 and 9.2:1, which are enough for a statistically meaningful analysis.

An initial refinement of the 100 K dataset yielded a model with large, elongated displacement ellipsoids at its periphery, and with several unrealistic C–C and C–N bond distances (Figure S10). There was also clear evidence of disorder of the methyl group on each ligand, about their two pyrazolyl *C*4 atom sites. These anomalies were addressed with a whole molecule disorder model. Two orientations were modelled for each ligand, with equivalent C–C and C–N bond lengths in each disorder site constrained to be the same. The occupancy ratio of the disorder sites for ligand N(2)-C(18) was refined as 0.60:0.40, while for ligand N(19)-C(35) it was 0.80:0.20; there is no reason why these should be the same in this monoclinic lattice symmetry.

The ligand disorder implies the iron atom should be disordered over four positions, with occupancies of 0.48, 0.32, 0.12 and 0.08. In practise three iron atom positions were identified in the Fourier map, and refined with occupancies of 0.50, 0.25 and 0.25. Both perchlorate ions are also disordered, and were modelled over two half-occupied orientations using refined Cl–O and O···O distance restraints. The U_{eq} displacement ellipsoids of the partial Cl atoms in one anion were also constrained to be the same. All non-H atoms with occupancy ≥0.5 in the complex cation, plus the half-occupied Cl atoms, were refined anisotropically. H atoms were placed in calculated positions and refined using a riding model.

The higher temperature datasets were treated the same way, starting from the whole molecule disorder model developed at 100 K. All fractional C atoms in the disorder model were refined isotropically at 300 K,

to maintain a reasonable observed data:parameter ratio at that temperature. The refinement residuals for the disordered cation model are clearly superior to the ordered cation model at 100, 180 and 220 K (Table S1). However at 300 K, the two models give essentially the same refinement statistics.

Two refinements are presented for each of these datasets, containing crystallographically ordered and whole-molecule-disordered complex cations. Metric parameters from the ordered cation models are useful for defining the spin state of the complex at each temperature, while the disordered cations give a better reflection of the true structure of the lattice.

Structure refinement of 3[BF$_4$]$_2$. This crystal is isomorphous with **1[ClO$_4$]$_2$**. The Br atom in each ligand is disordered about both possible pyrazolyl *C*4 atom sites. The occupancy ratio for Br(1A)/Br(1B) refined to 0.72:0.28, while for Br(3A)/Br(3B) it refined to 0.88:0.12. For simplicity, the main molecule in the refinement incorporates the major disorder site of each Br atom, with the minor Br atom sites being included as additional isolated partial atoms. The BF_4^- anions in the model are crystallographically ordered.

The refined Br disorder occupancies imply a 0.63:0.25:0.09:0.03 distribution of the four possible cation orientations in the crystal. Slightly high thermal parameters on other atoms at the periphery of the complex imply the Br atom disorder might be associated with whole molecule disorder of the cation. Attempts to refine a whole molecule disorder model of this structure were unsuccessful, however. That could reflect that this dataset only contains *ca* half the number of observed reflections as for **1[ClO$_4$]$_2$** at 100 K.

All non-H atoms except the 0.12-occupied Br atom site were refined anisotropically. H atoms were placed in calculated positions and refined using a riding model.

CCDC 2411009−2411013 contain the supplementary crystallographic data for this paper (Tables S1 and S2). These data can be obtained free of charge from The Cambridge Crystallographic Data Centre via www.ccdc.cam.ac.uk/data_request/cif

Other Measurements

Elemental analyses were performed by the microanalytical services at the London Metropolitan University School of Human Sciences. Electrospray mass spectra were recorded on a Bruker MicroTOF-q instrument from $CHCl_3$ solution. Diamagnetic NMR spectra employed a Bruker AV3HD spectrometer operating at 400.1 (^{1}H), 100.6 (^{13}C) or 376.5 MHz (^{19}F), while paramagnetic ^{1}H NMR spectra were obtained with a Bruker AV3 spectrometer operating at 300.1 MHz. Room temperature X-ray powder diffraction data were obtained with a Bruker D2 Phaser diffractometer using Cu-K_α radiation (λ = 1.5418 Å). Rietveld refinements of those powder patterns were performed using *TOPAS* v6,[7] starting from a structural model derived from the 300 K crystal structure of **1[ClO$_4$]$_2$**. The lattice parameters, crystal size broadening, background and scale factor were refined during the fits, with the sample displacement set at zero.

The variable temperature powder diffraction measurement of **2[ClO$_4$]$_2$** employed a Bruker D8-A25 instrument fitted with an Oxford Cryostream low-temperature device, in transmission capillary geometry with a Ge(111) Johanssen monochromator and a Lynxeye detector. The other variable temperature powder diffraction studies were performed with a Huber G670 Guinier camera equipped with a Germanium monochromator and an imaging plate detector, operating in transmission geometry. A thin layer of the sample was placed between two Mylar films and clamped onto a sample holder designed for the cryogenic setup on the diffractometer. The sample was cooled to 15 K using a closed-loop helium cryojet, and the temperature was stabilized at 15 K for one hour before measurements. The samples were poised at each temperature for 5 mins before measurement, and data from an empty sample holder were subtracted as a background correction. Both powder diffractometers used Cu-K_α radiation (λ = 1.5418 Å).

Magnetic susceptibility measurements were performed using a Quantum Design MPMS-3 VSM magnetometer, in an applied field of 5000 G. Diamagnetic corrections for the samples were estimated from Pascal's constants;[8] a previously measured diamagnetic correction for the sample holder was also applied to the data. Data were collected with cooling and warming temperature ramps, at a scan rate of 2 K min^{-1}. Differential scanning calorimetry (DSC) measurements were performed with a TA Instruments Q2000 heat flux calorimeter with a TA RCS90 refrigerated cooling system. The samples were prepared in aluminium, hermetically sealed pans and scanned at a rate of 5 K min^{-1}.

Table S1 Crystallographic experimental data for **1[ClO$_4$]$_2$**. Two values for each refinement residual are given, for the cation disorder model and [in square brackets] the crystallographically ordered cation refinements. These data were collected using synchrotron radiation.

T / K	100(2)	180(2)	220(2)	300(2)
Spin state	low-spin	low-spin	predominantly low-spin	predominantly high-spin
formula	$C_{24}H_{22}Cl_2FeN_{10}O_8$			
M_r	705.26			
crystal class	monoclinic			
space group	$P2_1$			
Z	2			
a / Å	9.0570(3)	9.0959(2)	9.1111(2)	9.0949(10)
b / Å	9.1234(2)	9.1543(2)	9.1607(2)	9.0814(7)
c / Å	18.0236(4)	18.0900(3)	18.1574(3)	18.7450(14)
α / deg	90	90	90	90
β / deg	97.553(2)	97.453(2)	97.194(2)	93.755(7)
γ / deg	90	90	90	90
V / Å^3	1476.38(7)	1493.57(5)	1503.56(5)	1544.9(2)
μ / mm^{-1}	0.700	0.692	0.687	0.669
D_c / gcm^{-3}	1.586	1.568	1.558	1.516
measured reflections	18774	19007	19174	17566
independent reflections	7684 [*7686*]	7765 [*7770*]	7835 [*7840*]	6770 [*6778*]
R_{int}	0.039 [*0.039*]	0.036 [*0.036*]	0.037 [*0.037*]	0.055 [*0.055*]
parameters	565 [*438*]	565 [*438*]	564 [*438*]	444 [*438*]
restraints	193 [*31*]	193 [*31*]	193 [*31*]	193 [*31*]
R_1 [$F_0 > 4\sigma(F_0)$][a]	0.056 [*0.076*]	0.053 [*0.070*]	0.055 [*0.068*]	0.072 [*0.066*]
wR_2, all data[b]	0.169 [*0.231*]	0.165 [*0.236*]	0.178 [*0.228*]	0.242 [*0.224*]
goodness of fit	1.053 [*1.109*]	0.930 [*1.047*]	0.921 [*1.040*]	0.866 [*0.865*]
$\Delta\rho_{min/max}$ / eÅ^{-3}	–0.46/0.42 [*–0.97/0.79*]	–0.44/0.33 [*–0.93/0.53*]	–0.39/0.30 [*–0.78/0.38*]	–0.41/0.46 [*–0.40/0.46*]
Flack parameter	–0.004(14) [*–0.003(16)*]	–0.006(14) [*0.00(3)*]	–0.005(16) [*0.001(16)*]	0.05(2) [*0.05(2)*]
CCDC[c]	2411009	2411010	2411011	2411012

[a] $R = \Sigma[|F_o| - |F_c|] / \Sigma|F_o|$ [b] $wR = [\Sigma w(F_o^2 - F_c^2) / \Sigma wF_o^4]^{1/2}$ [c] The disordered cation refinements were deposited with the CCDC.

Table S2 Crystallographic experimental data for the other complexes in this work, including unit cell data for compounds that diffracted too weakly for a full structure determination. The data were collected using synchrotron radiation unless otherwise stated.

	**2[BF$_4$]$_2$**	**3[BF$_4$]$_2$**		[Fe(L^{Cl})$_2$][BF$_4$]$_2$
T / K	100(2)	100(2)	300(2)	200(2)
Spin state	Low-spin[c]	Low-spin	High-spin[c]	Low-spin[c]
formula	$C_{22}H_{16}B_2F_{10}FeN_{10}$	$C_{22}H_{16}B_2Br_2F_8FeN_{10}$		$C_{22}H_{16}B_2Cl_2F_8FeN_{10}$
M_r	685.90	809.74		720.82
crystal class	monoclinic	monoclinic		monoclinic
space group	$P2_1$	$P2_1$		$P2_1$
Z	2	2		2
a / Å	9.095(1)	9.5177(12)	9.1683(6)	9.245(3)
b / Å	8.767(3)	9.3657(13)	9.1687(7)	9.216(2)
c / Å	18.123(5)	16.524(3)	18.2513(15)	17.225(6)
α / deg	90	90	90	90
β / deg	98.22(3)	93.848(13)	91.303(7)	94.30(4)
γ / deg	90	90	90	90
V / Å^3	1370.0(6)	1469.7(4)	1533.8(2)	1463.4(8)
μ / mm^{-1}	0.604	3.046	2.918	6.596[d]
D_c / gcm^{-3}	1.663	1.830	1.753	1.636
measured reflections	–	16061	–	–
independent reflections	–	6420	–	–
R_{int}	–	0.071	–	–
parameters	–	419	–	–
restraints	–	1	–	–
R_1 [$F_0 > 4\sigma(F_0)$][a]	–	0.050	–	–
wR_2, all data[b]	–	0.108	–	–
goodness of fit	–	0.765	–	–
$\Delta\rho_{min/max}$ / eÅ^{-3}	–	–0.68/0.55	–	–
Flack parameter		0.024(16)	–	
CCDC	–	2411013	–	–

[a]$R = \Sigma[|F_o| - |F_c|] / \Sigma|F_o|$ [b]$wR = [\Sigma w(F_o^2 - F_c^2) / \Sigma wF_o^4]^{1/2}$ [c]This dataset diffracted too weakly for a full structure solution and refinement.
[d]Collected with Cu-K_α radiation (λ = 1.5418 Å).

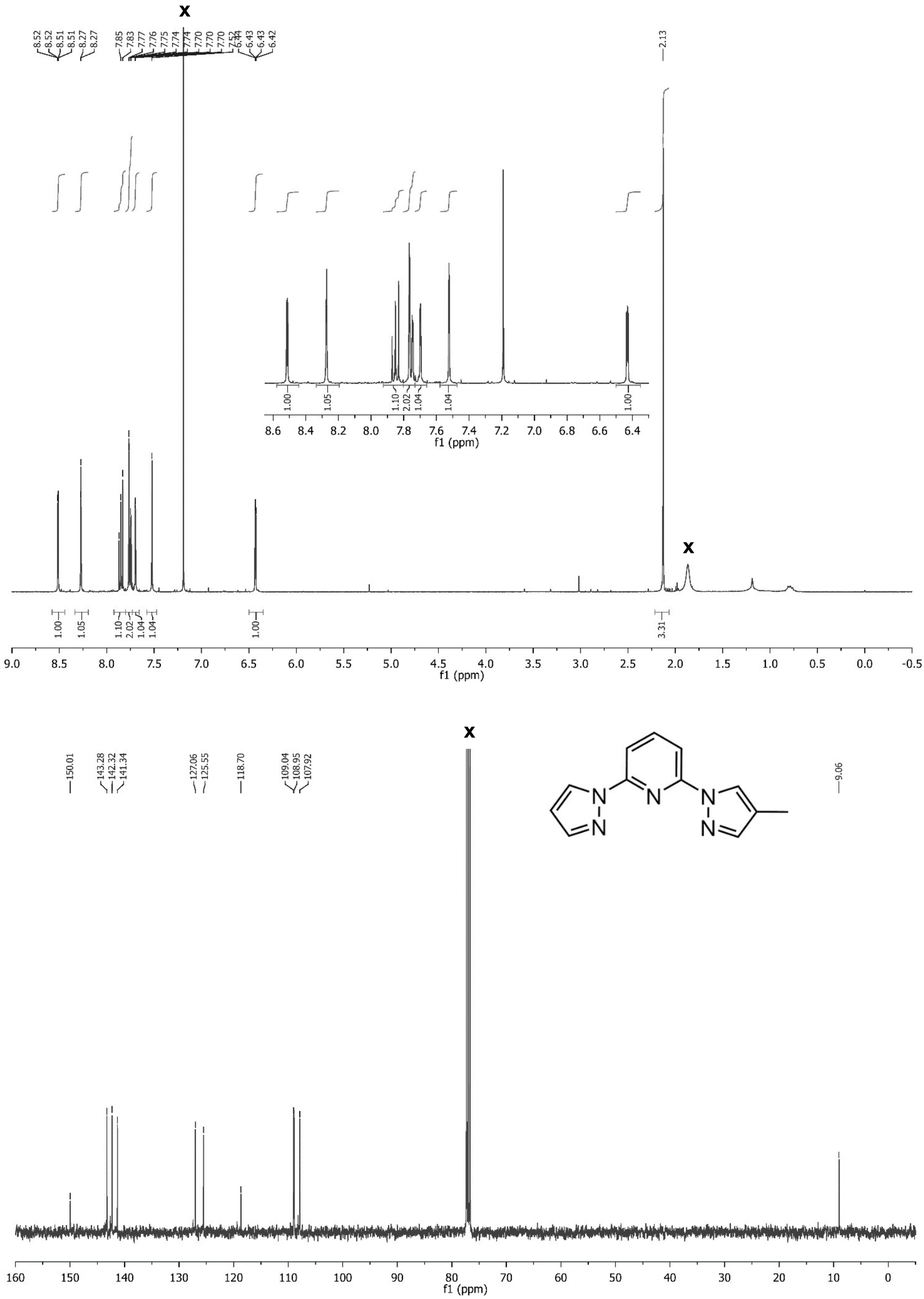

Figure S1 ^{1}H (top) and ^{13}C (bottom) NMR spectra of L^{Me} ($CDCl_3$)

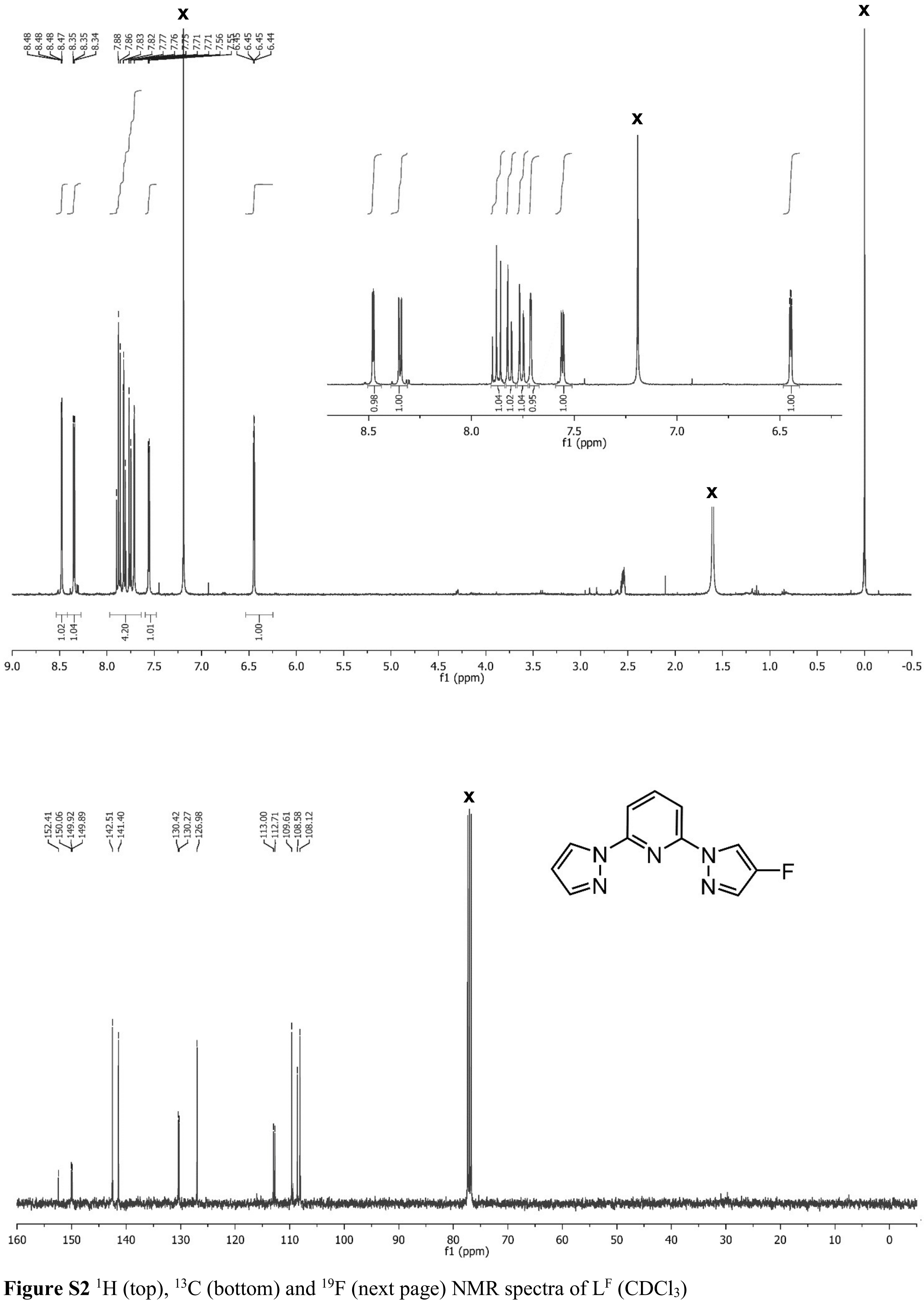

Figure S2 ^{1}H (top), ^{13}C (bottom) and ^{19}F (next page) NMR spectra of L^F ($CDCl_3$)

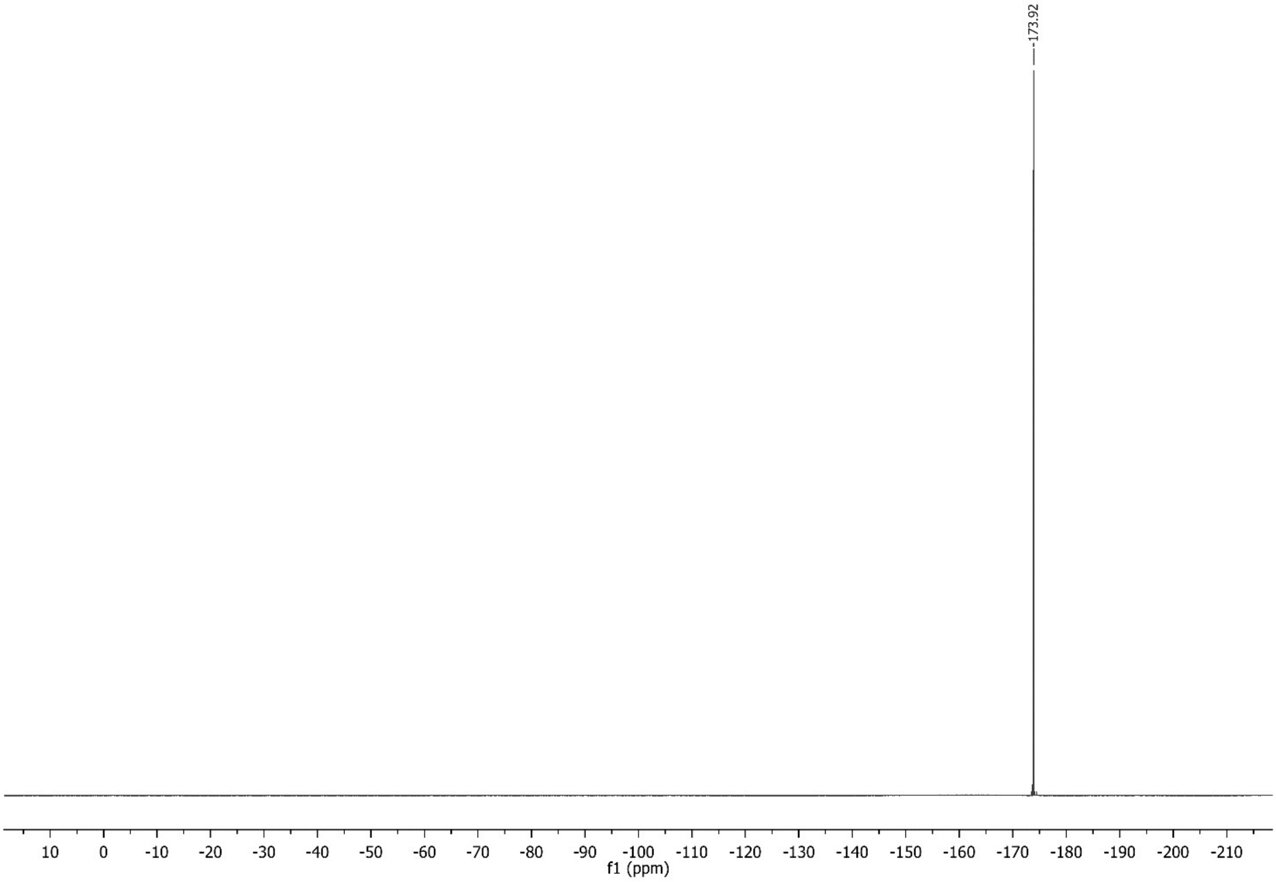

Figure S2 continued.

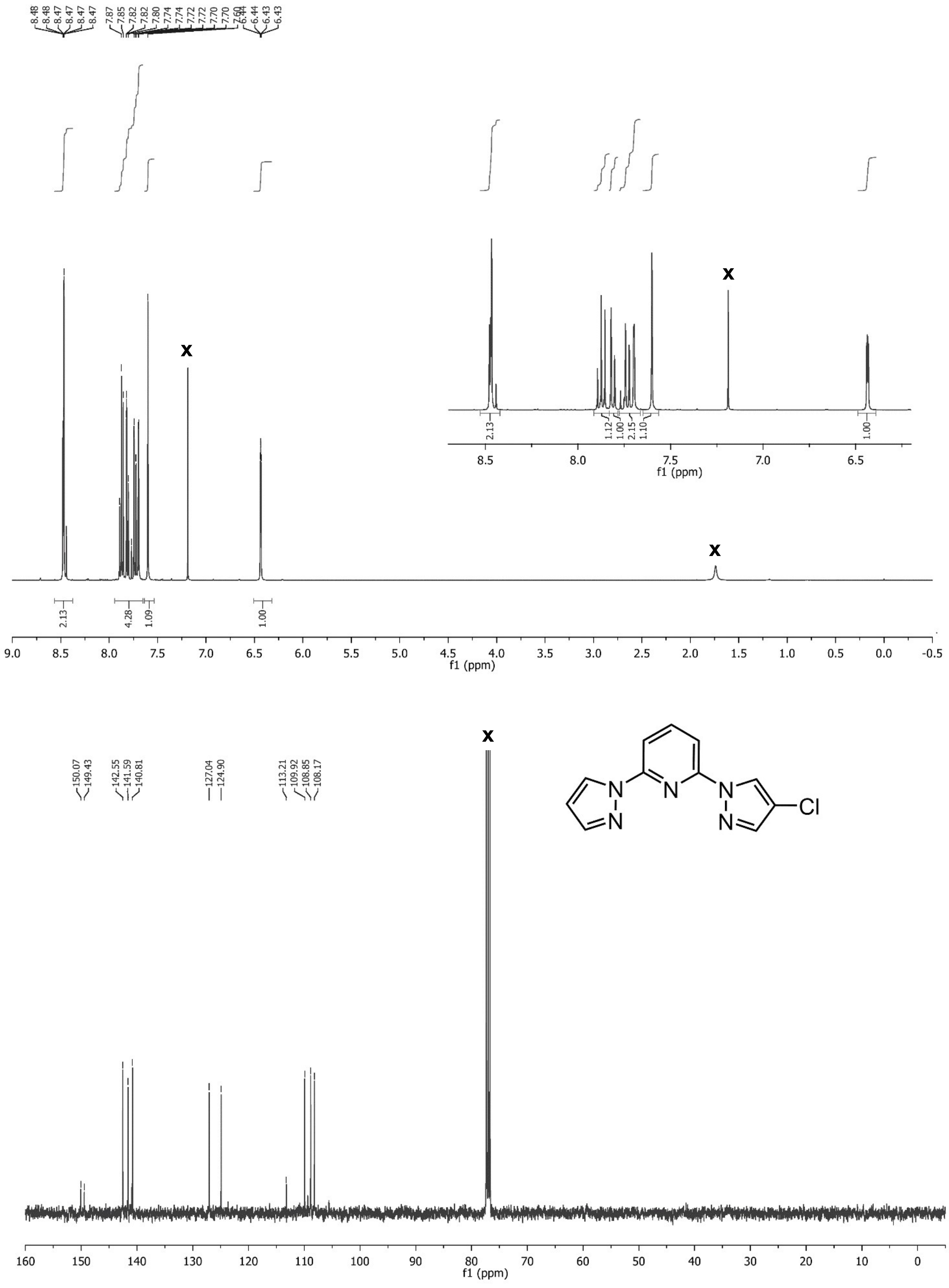

Figure S3 ^{1}H (top) and ^{13}C (bottom) NMR spectra of L^{Cl} ($CDCl_3$)

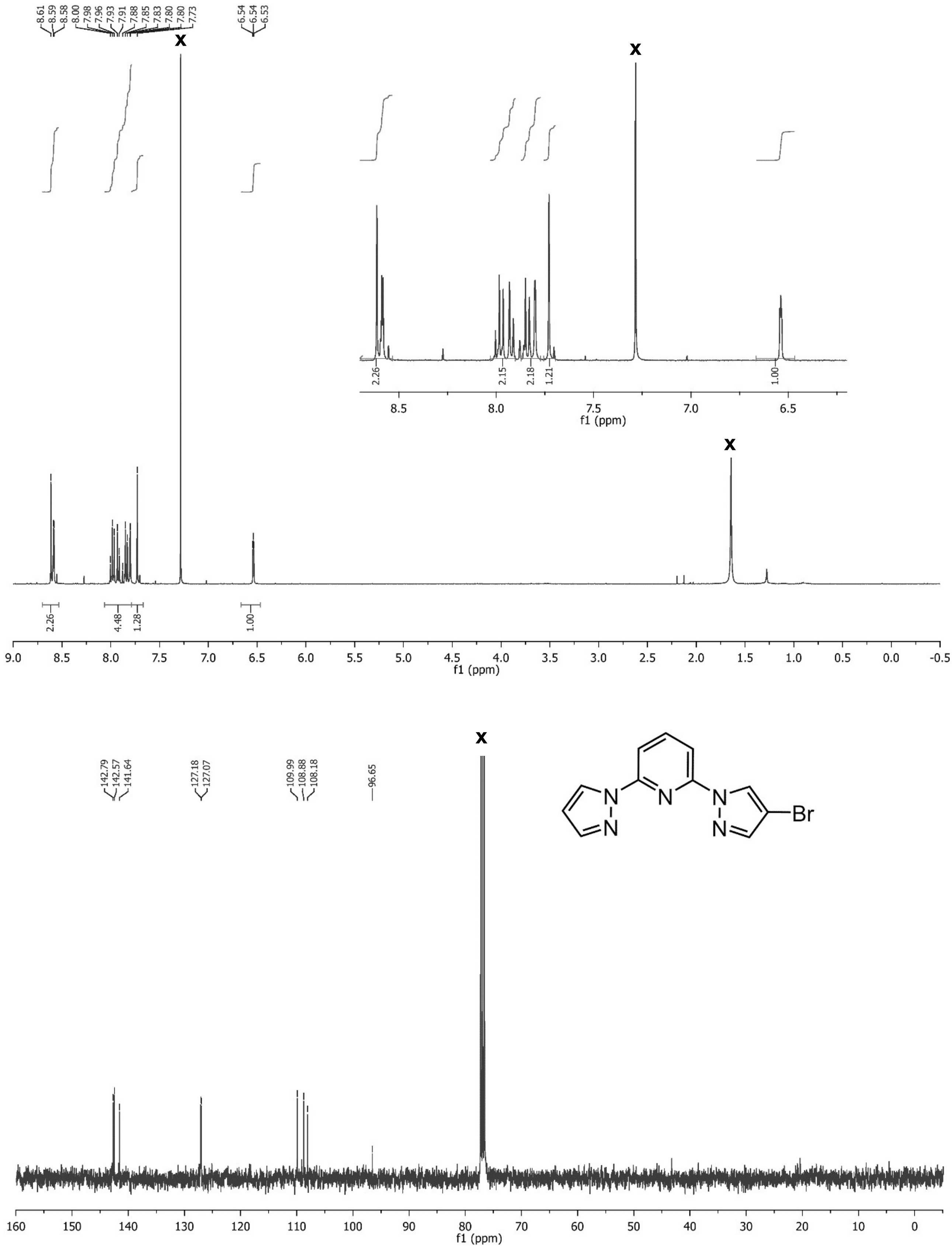

Figure S4 ^{1}H (top) and ^{13}C (bottom) NMR spectra of L^{Br} ($CDCl_3$).

The quaternary Py C^2 and C^6 ^{13}C resonances near 150 ppm were not observed for this compound, reflecting its lower solubility (*cf* Figure S3).

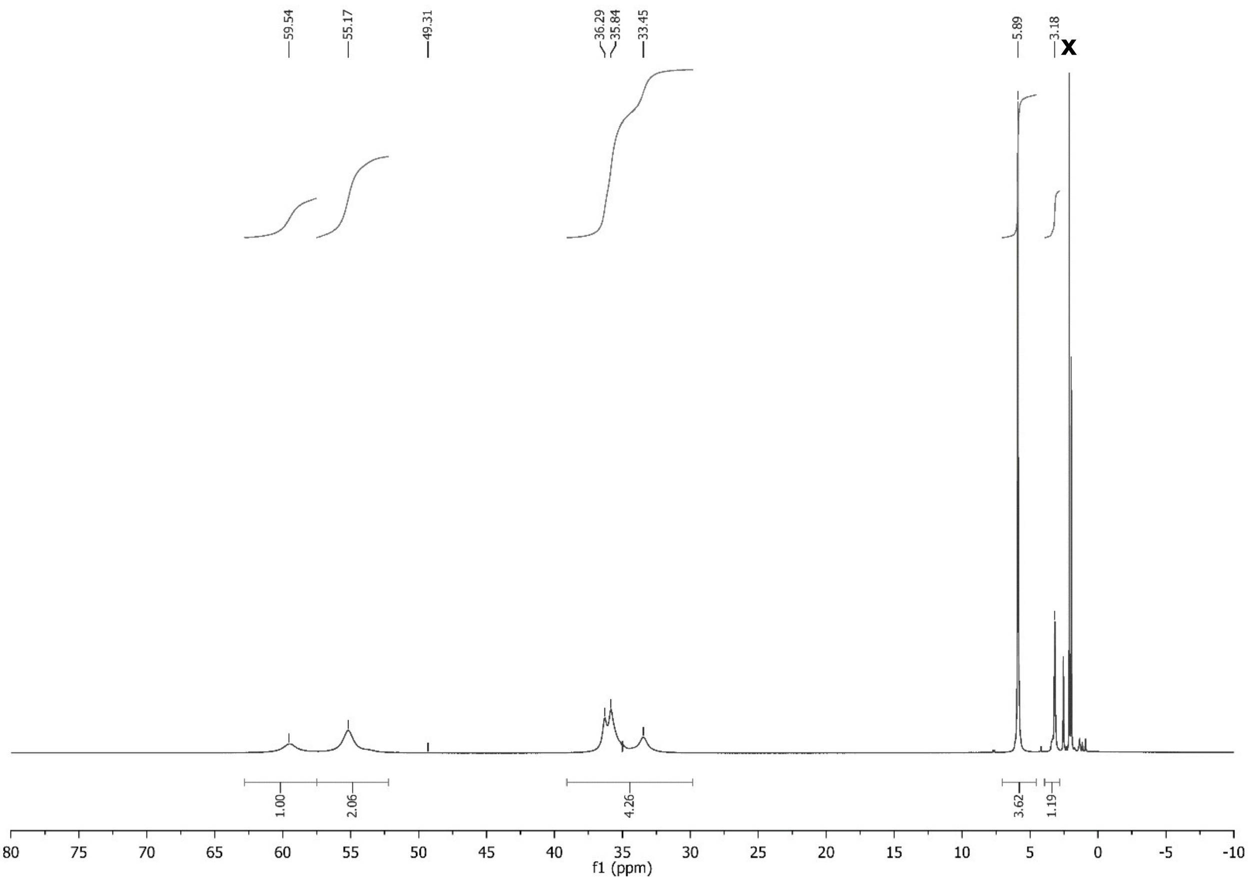

Figure S5 Paramagnetic ^{1}H NMR spectrum of **1[BF$_4$]$_2$** (CD_3CN).

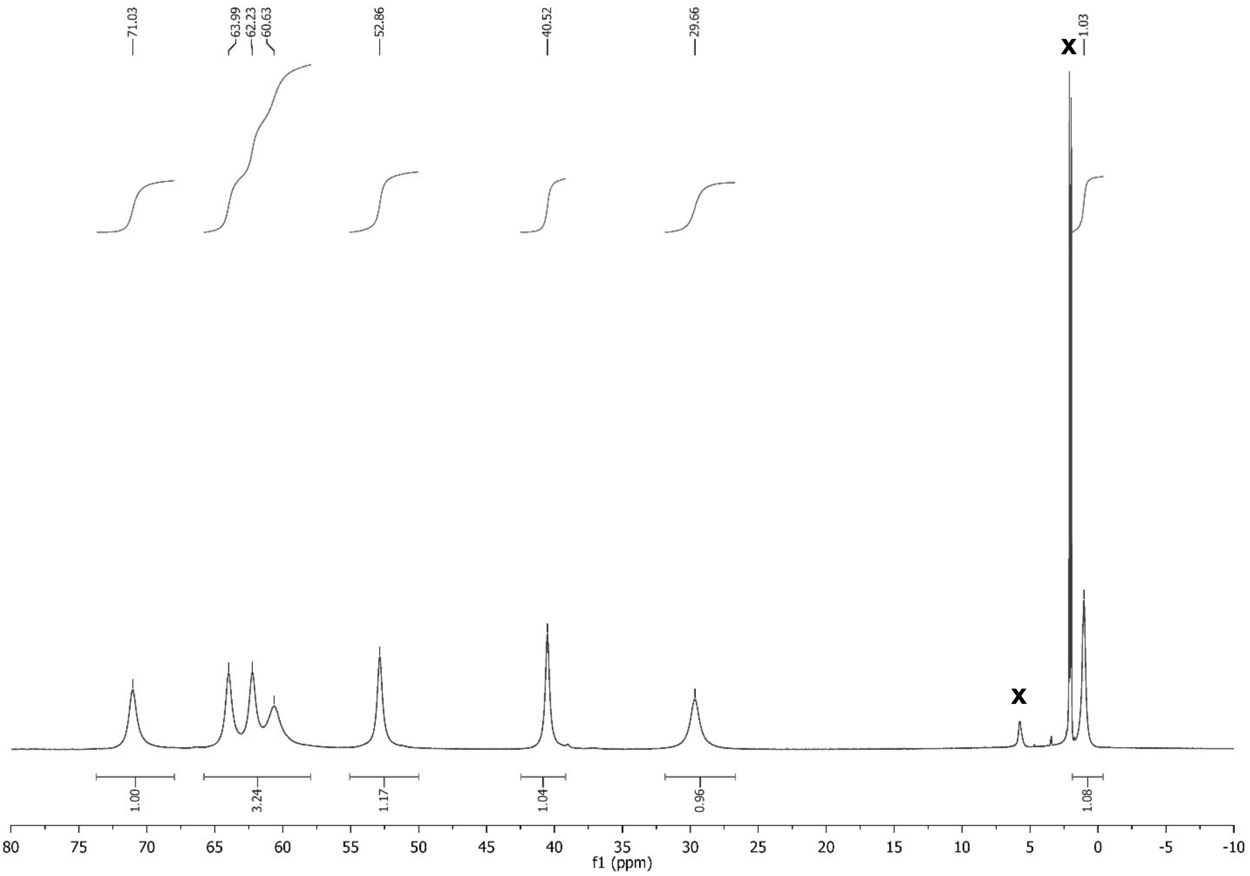

Figure S6 Paramagnetic ^{1}H NMR spectrum of **2[BF$_4$]$_2$** (CD_3CN).

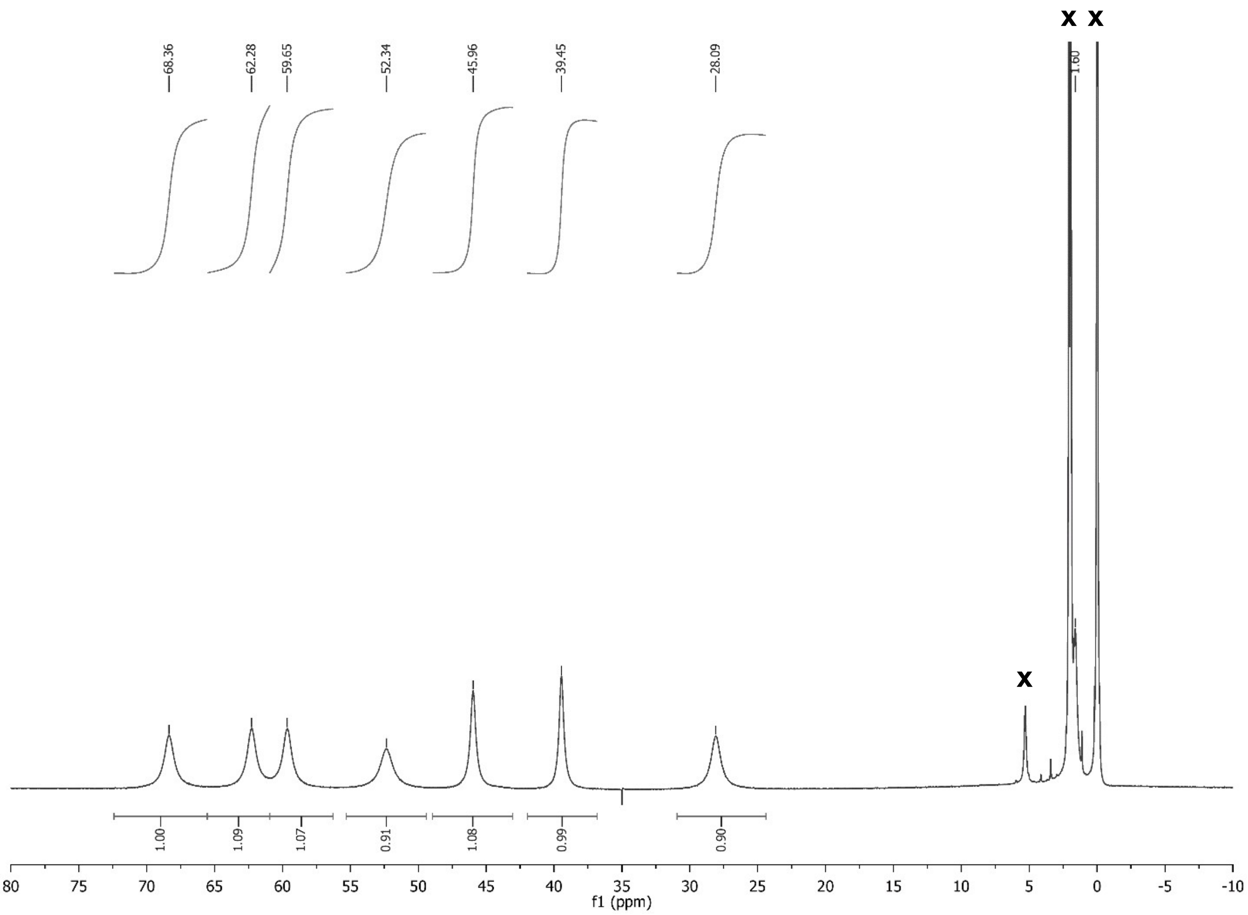

Figure S7 Paramagnetic ^{1}H NMR spectrum of **3[BF4]2** (CD_3CN).

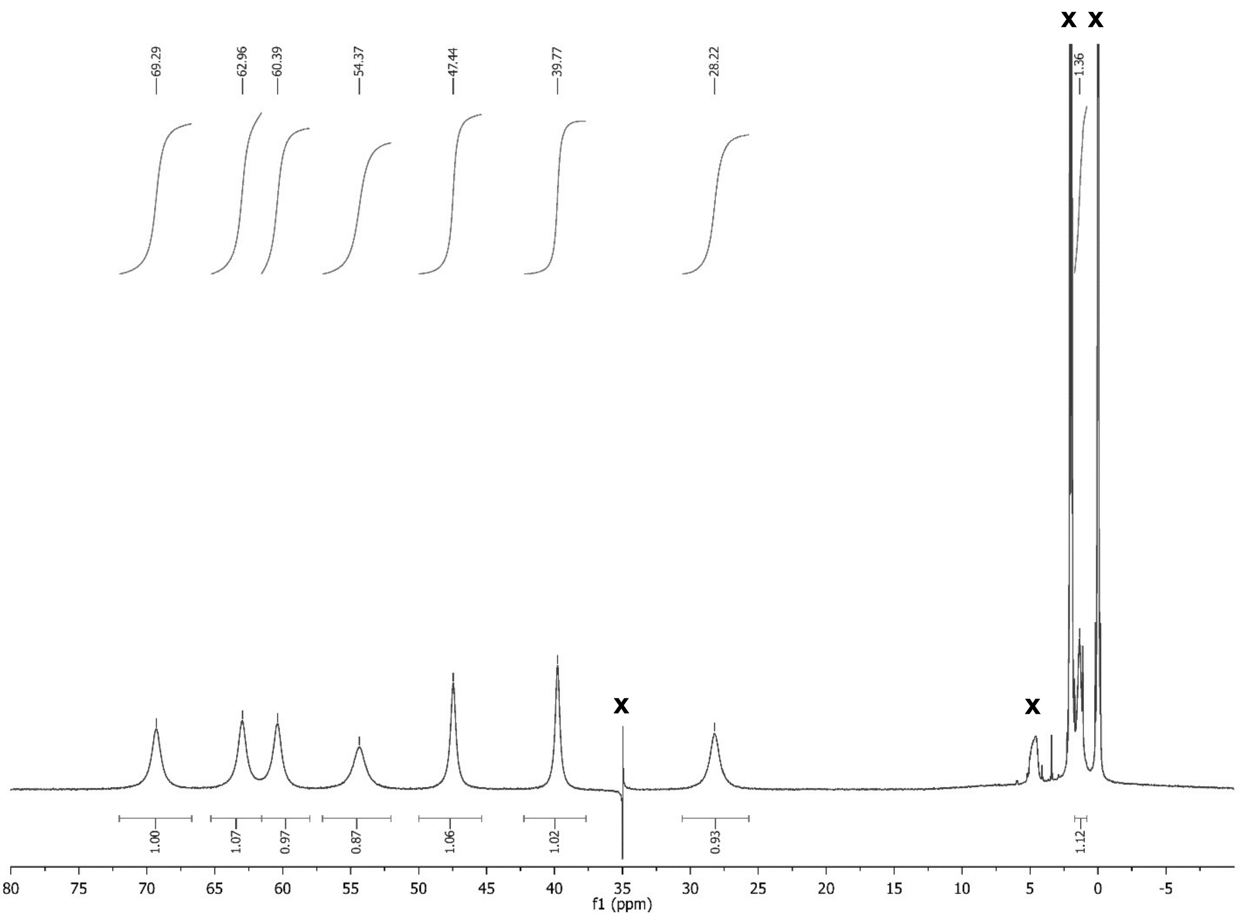

Figure S8 Paramagnetic ^{1}H NMR spectrum of $[Fe(L^{Cl})_2][BF_4]_2$ (CD_3CN).

Definitions of the structural parameters discussed in the paper.

V_{Oh} is the volume (in Å^3) of the FeN_6 coordination octahedron in the complex,[9] which is typically <10 Å^3 in low-spin $[Fe(bpp)_2]^{2+}$ (bpp = 2,6-di{pyrazol-1-yl}pyridine) derivatives and ≥11.5 Å^3 in their high-spin form.[10]

Σ and Θ are defined as follows:

$$\Sigma = \sum_{i=1}^{12} |90 - \beta_i| \qquad \Theta = \sum_{j=1}^{24} |60 - \gamma_j|$$

where β_i are the twelve *cis*-N–Fe–N angles about the iron atom and γ_i are the 24 unique N–Fe–N angles measured on the projection of two triangular faces of the octahedron along their common pseudo-threefold axis (Scheme S1). Σ is a general measure of the deviation of a metal ion from an ideal octahedral geometry, while Θ more specifically indicates its distortion towards a trigonal prismatic structure. A perfectly octahedral complex gives $\Sigma = \Theta = 0$.[9,11]

Because the high-spin state of a complex has a much more plastic structure than the low-spin, this is reflected in Σ and Θ which are usually much larger in the high-spin state. The absolute values of these parameters depend on the metal/ligand combination in the compound under investigation, however. Typical values of these parameters for complexes related to $[Fe(bpp^R)_2]^{2+}$ are given in refs. 12 and 13.

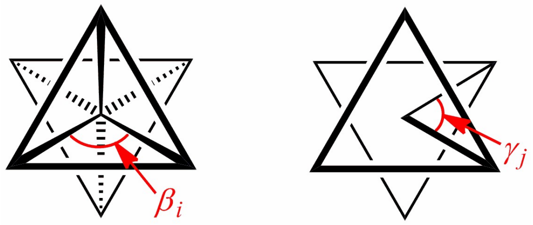

Scheme S1 Angles used in the definitions of the coordination distortion parameters Σ and Θ.

The parameters in Scheme S2 define the magnitude of an angular Jahn-Teller distortion, that is often observed in high-spin $[Fe(bpp)_2]^{2+}$ derivatives like $[FeL_2]^{2+}$ ($\theta \leq 90$°, $\phi \leq 180$ °).[13,14] They are also a useful indicator of the molecular geometry, in defining the disposition of the two ligands around the metal ion. Spin-crossover can be inhibited if θ and ϕ deviate too strongly from their ideal values, because the associated rearrangement to a more regular low-spin coordination geometry ($\theta \approx 90$°, $\phi \approx 180$°) cannot be accommodated by a rigid solid lattice.[14-16] However, in less distorted examples, significant changes in θ and ϕ between the spin states can be associated with enhanced SCO cooperativity.[17-20]

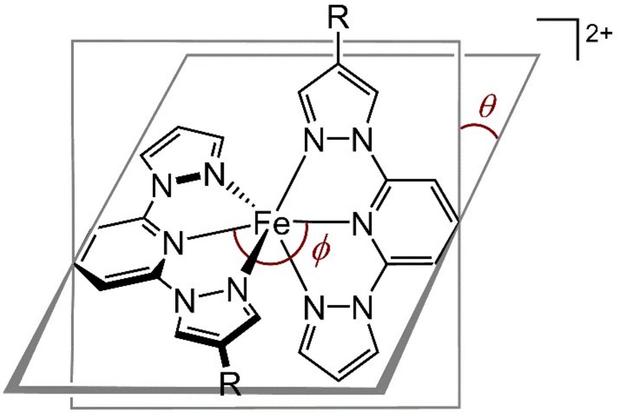

Scheme S2 θ and ϕ, used to discuss the structures in this work.

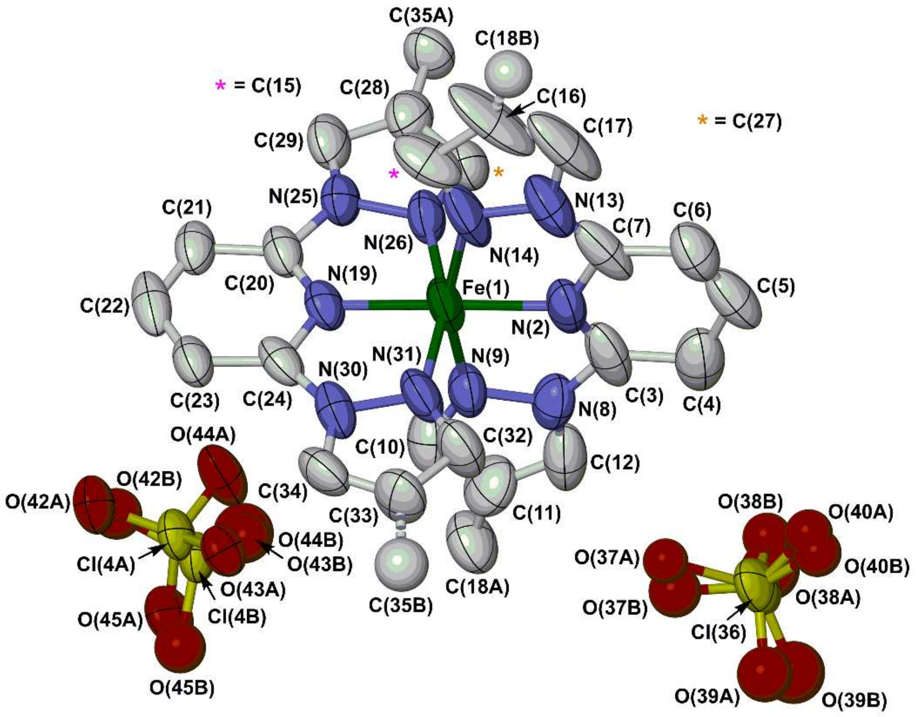

Figure S9 The asymmetric unit of the ordered cation refinement of $[Fe(L^{Me})_2][ClO_4]_2$ (**1[ClO$_4$]$_2$**) at 100 K, showing the full atom numbering scheme. The (anisotropic) major and (isotropic) minor disorder sites for the methyl C atoms, and both orientations of each disordered anion, are shown. Atomic displacement ellipsoids are at the 50 % probability level, while H atoms have been omitted for clarity.

Colour code: C, white; Cl, yellow; Fe, green; N, blue; O, cyan.

The atom numbering in the disordered cation refinements of this compound follows this scheme, but with 'A', 'B' or 'C' suffixes on each label to distinguish the disorder sites of each residue in the molecule

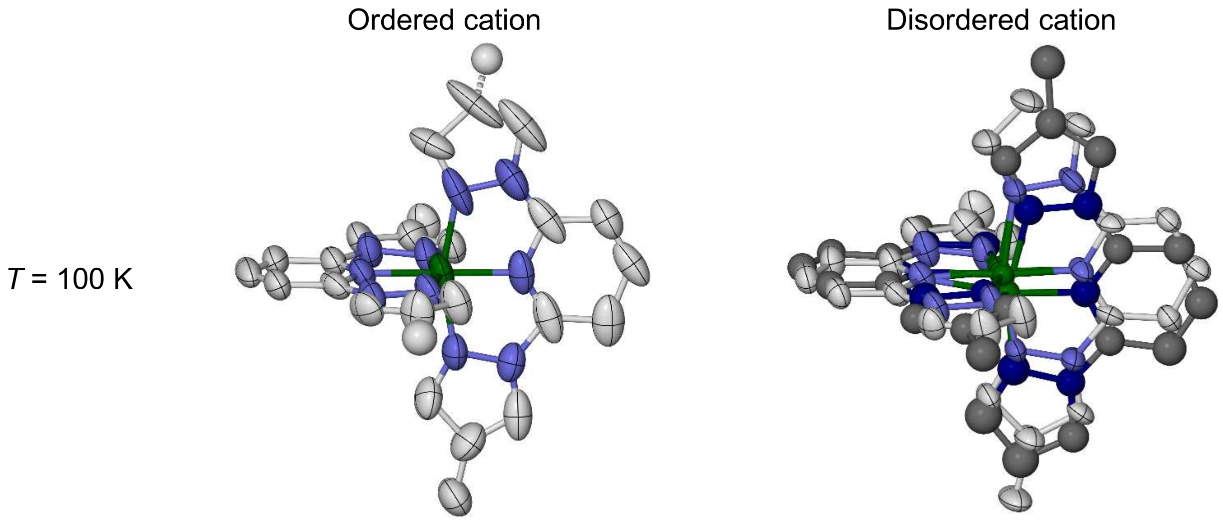

Figure S10 Comparison of the ordered and disordered cation refinements of **1[ClO$_4$]$_2$** at each temperature. The major ('A') and minor ('B') orientations of each ligand in the disordered cations are shown with pale and dark colouration, respectively. Three partial Fe atom sites (out of a possible four) are also included in the disordered model. Other details as for Figure S9.

Colour code: C, white or dark gray; Fe, green; N, pale or dark blue.

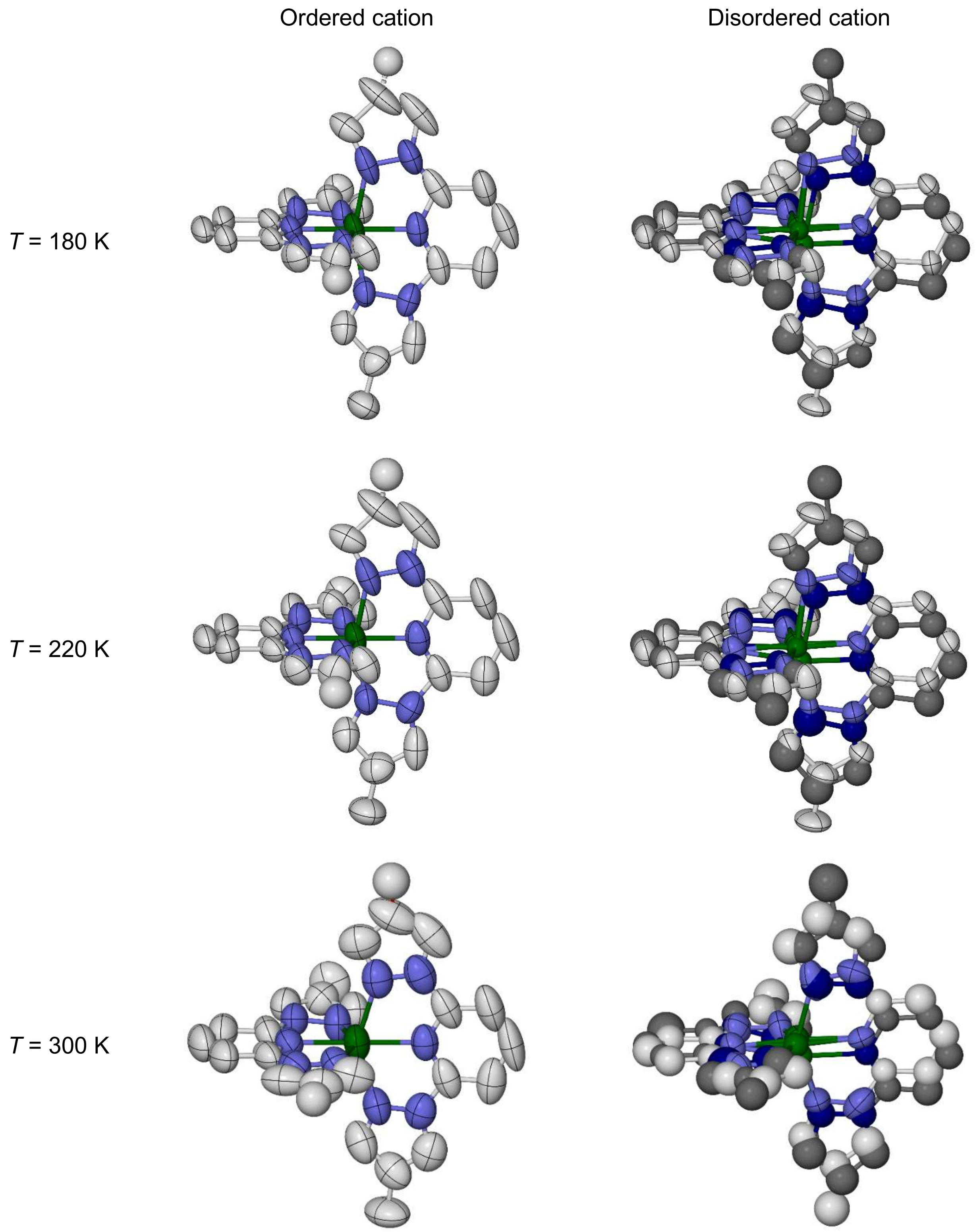

Figure S10 continued.

The disordered cation refinements at 100, 180 and 220 K give significantly lower refinement residuals (Table S1), and have more reasonable bond lengths and angles within the L^{Me} ligands. The ordered and disordered cation models give essentially identical residuals at 300 K, but the orientational disorder that is clearly present at 100 K should still occur at room temperature.

Table S3 Selected bond lengths, angles and structural parameters (Å , deg, Å[3]) for the ordered cation refinements of **1[ClO$_4$]$_2$**. See Figure S9 for the atom numbering scheme, while definitions of V_{Oh}, Σ, Θ, φ and θ are given on page S15.

T	300 K	220 K	180 K	100 K
Fe(1)–N(2)	2.118(6)	1.906(6)	1.900(6)	1.886(7)
Fe(1)–N(9)	2.173(8)	1.961(7)	1.964(7)	1.954(8)
Fe(1)–N(14)	2.179(10)	2.000(10)	2.011(10)	2.010(10)
Fe(1)–N(19)	2.112(6)	1.911(5)	1.899(5)	1.896(6)
Fe(1)–N(26)	2.173(8)	1.978(7)	1.966(7)	1.967(8)
Fe(1)–N(31)	2.208(9)	2.002(8)	1.990(8)	1.993(9)
N(2)–Fe(1)–N(9)	74.2(3)	81.2(3)	81.2(3)	81.3(4)
N(2)–Fe(1)–N(14)	72.0(3)	78.2(3)	79.0(3)	79.1(4)
N(2)–Fe(1)–N(19)	177.3(4)	178.1(3)	178.4(3)	178.4(4)
N(2)–Fe(1)–N(26)	109.2(3)	101.4(3)	101.0(3)	100.9(3)
N(2)–Fe(1)–N(31)	104.0(3)	98.9(3)	98.6(3)	98.6(3)
N(9)–Fe(1)–N(14)	146.2(3)	159.3(3)	160.1(3)	160.3(3)
N(9)–Fe(1)–N(19)	106.5(2)	99.8(2)	99.4(2)	99.1(3)
N(9)–Fe(1)–N(26)	94.3(3)	90.9(3)	90.4(3)	90.4(3)
N(9)–Fe(1)–N(31)	94.0(3)	92.1(3)	91.7(3)	91.8(3)
N(14)–Fe(1)–N(19)	107.3(3)	100.8(3)	100.5(3)	100.5(3)
N(14)–Fe(1)–N(26)	95.2(3)	91.9(3)	91.9(3)	92.0(3)
N(14)–Fe(1)–N(31)	95.5(3)	92.3(3)	92.7(3)	92.4(3)
N(19)–Fe(1)–N(26)	73.4(3)	80.2(3)	80.5(3)	80.7(3)
N(19)–Fe(1)–N(31)	73.5(3)	79.5(3)	79.9(3)	79.8(3)
N(26)–Fe(1)–N(31)	146.8(3)	159.7(2)	160.4(3)	160.5(3)
V_{Oh}	12.32(3)	9.71(2)	9.66(2)	9.61(3)
Σ	152.9(10)	89.0(10)	85.6(10)	84.8(11)
Θ	502	293	282	279
φ	177.3(4)	178.1(3)	178.4(3)	178.4(4)
θ	89.99(7)	89.50(7)	89.39(7)	89.28(7)

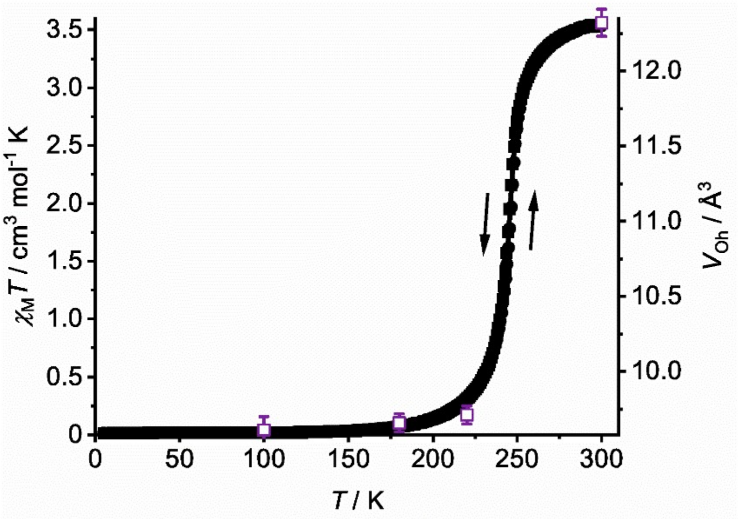

Figure S11 Comparison of the spin-transition in **1[ClO$_4$]$_2$** as measured by variable temperature magnetic susceptibility data (●) and from its ordered cation crystallographic refinements (Table S3; □). The bond length parameter V_{Oh} is defined on page S15, and the error bars on V_{Oh} correspond to 3σ.

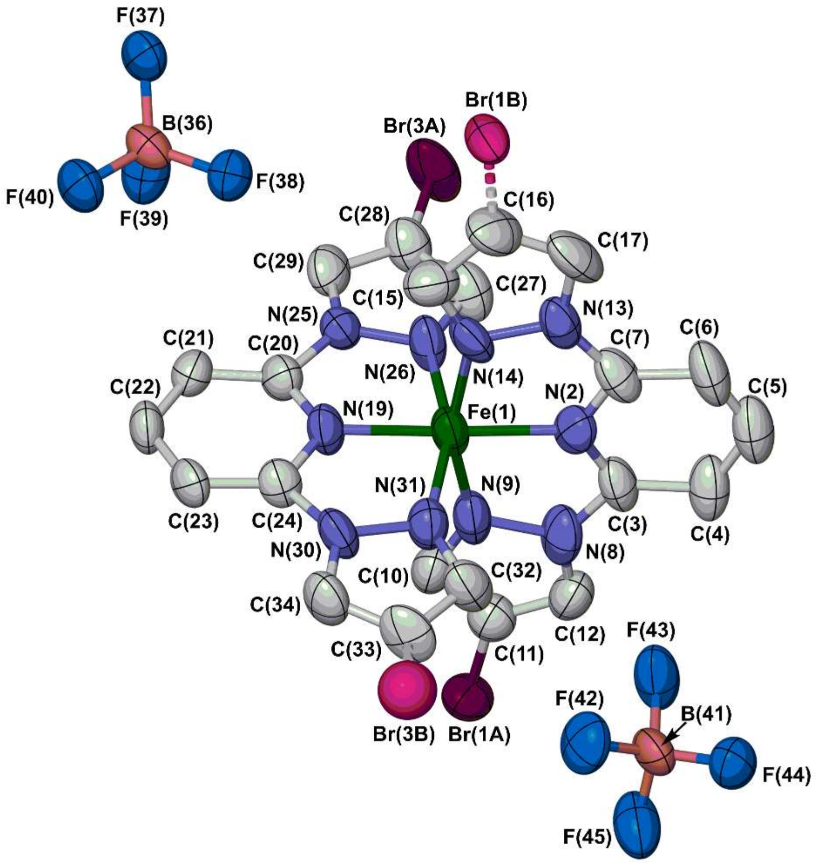

Figure S12 The asymmetric unit of $[Fe(L^{Br})_2][BF_4]_2$ (**3[BF₄]₂**) at 100 K, showing the full atom numbering scheme. The major and minor disorder sites for each Br atom are shown with dark and pale colouration, respectively. Other details as for Figure S9.

Colour code: C, white; B, pink; Br, dark or pale maroon; F, cyan; Fe, green; N, blue.

Table S4 Selected bond lengths, angles and structural parameters (Å , deg, Å^3) for low-spin **$3[BF_4]_2$** at 100 K. See Figure S12 for the atom numbering scheme. Other details as for Table S3.

Fe(1)–N(2)	1.904(7)	Fe(1)–N(19)	1.891(6)
Fe(1)–N(9)	1.959(10)	Fe(1)–N(26)	1.943(7)
Fe(1)–N(14)	1.981(9)	Fe(1)–N(31)	1.966(7)
N(2)–Fe(1)–N(9)	81.1(4)	N(9)–Fe(1)–N(31)	92.0(3)
N(2)–Fe(1)–N(14)	79.5(4)	N(14)–Fe(1)–N(19)	97.9(3)
N(2)–Fe(1)–N(19)	177.3(4)	N(14)–Fe(1)–N(26)	91.3(3)
N(2)–Fe(1)–N(26)	100.0(3)	N(14)–Fe(1)–N(31)	94.1(3)
N(2)–Fe(1)–N(31)	100.0(3)	N(19)–Fe(1)–N(26)	80.3(3)
N(9)–Fe(1)–N(14)	160.4(3)	N(19)–Fe(1)–N(31)	79.9(3)
N(9)–Fe(1)–N(19)	101.6(3)	N(26)–Fe(1)–N(31)	160.0(3)
N(9)–Fe(1)–N(26)	89.3(4)		
V_{Oh}	9.45(2)	φ	177.3(4)
Σ	86.8(11)	θ	89.67(7)
Θ	281		

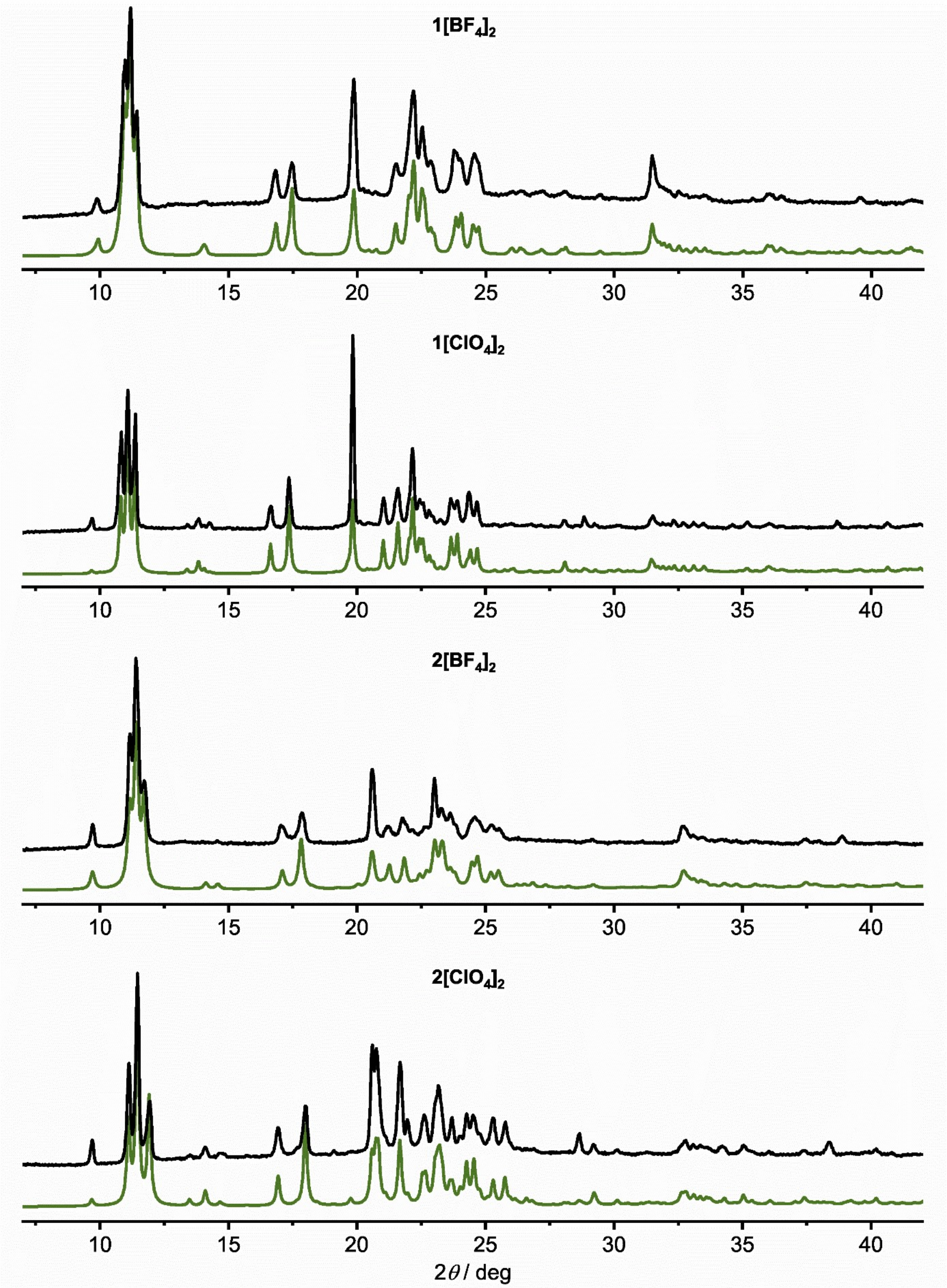

Figure S13 Measured (black) and simulated (green) room temperature X-ray powder diffraction data for the complexes in this study.

Simulations are Rietveld refinements of the unit cell parameters. Small differences between the measured and simulated peak intensities reflect preferred orientation effects in the samples.

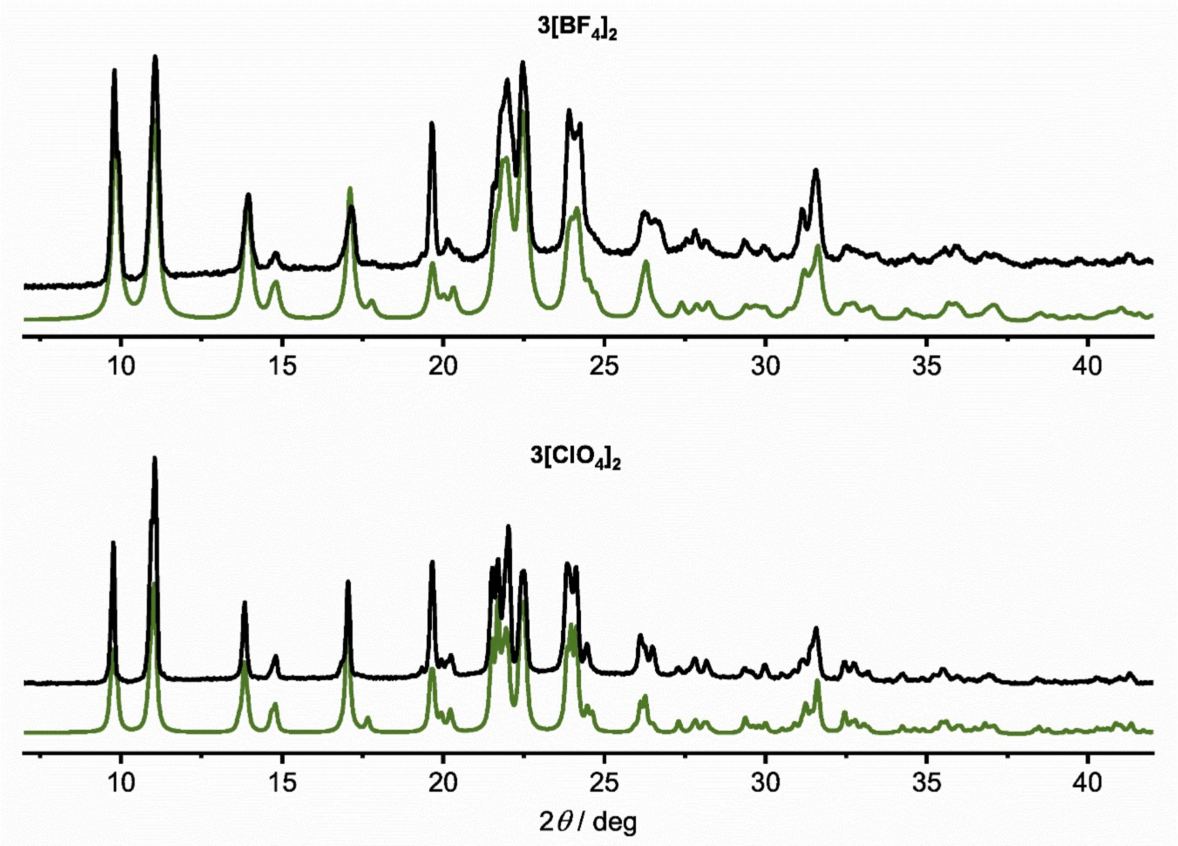

Figure S13 continued.

Table S5 Unit cell parameters from Rietveld refinements of the room temperature powder diffraction data (Figure S13).

	a / Å	*b* / Å	*c* / Å	α / deg	β / deg	γ / deg	V / Å^3
**1[BF$_4$]$_2$**	9.070(4)	9.070(4)	18.375(10)	90	93.17(3)	90	1509.3(12)
**1[ClO$_4$]$_2$**[a]	9.063(2)	9.054(2)	18.743(5)	90	93.701(13)	90	1534.8(6)
**2[BF$_4$]$_2$**	8.717(8)	8.725(8)	18.656(17)	90	93.70(2)	90	1416(2)
**2[ClO$_4$]$_2$**	8.740(4)	8.765(4)	18.995(9)	90	95.414(19)	90	1448.7(11)
**3[BF$_4$]$_2$**[a]	9.141(9)	9.120(8)	18.435(18)	90	91.25(3)	90	1537(3)
**3[ClO$_4$]$_2$**	9.149(2)	9.108(2)	18.563(5)	90	91.108(15)	90	1546.5(7)
[Fe(L^{Cl})$_2$[BF$_4$]$_2$[b]	9.006(6)	9.015(6)	18.220(12)	90	91.349(18)	90	1478.9(17)

[a]These parameters are consistent with the single crystal unit cell of this compound (Tables S1 and S2).
[b]The powder pattern for this compound is shown in Figure S21 below.

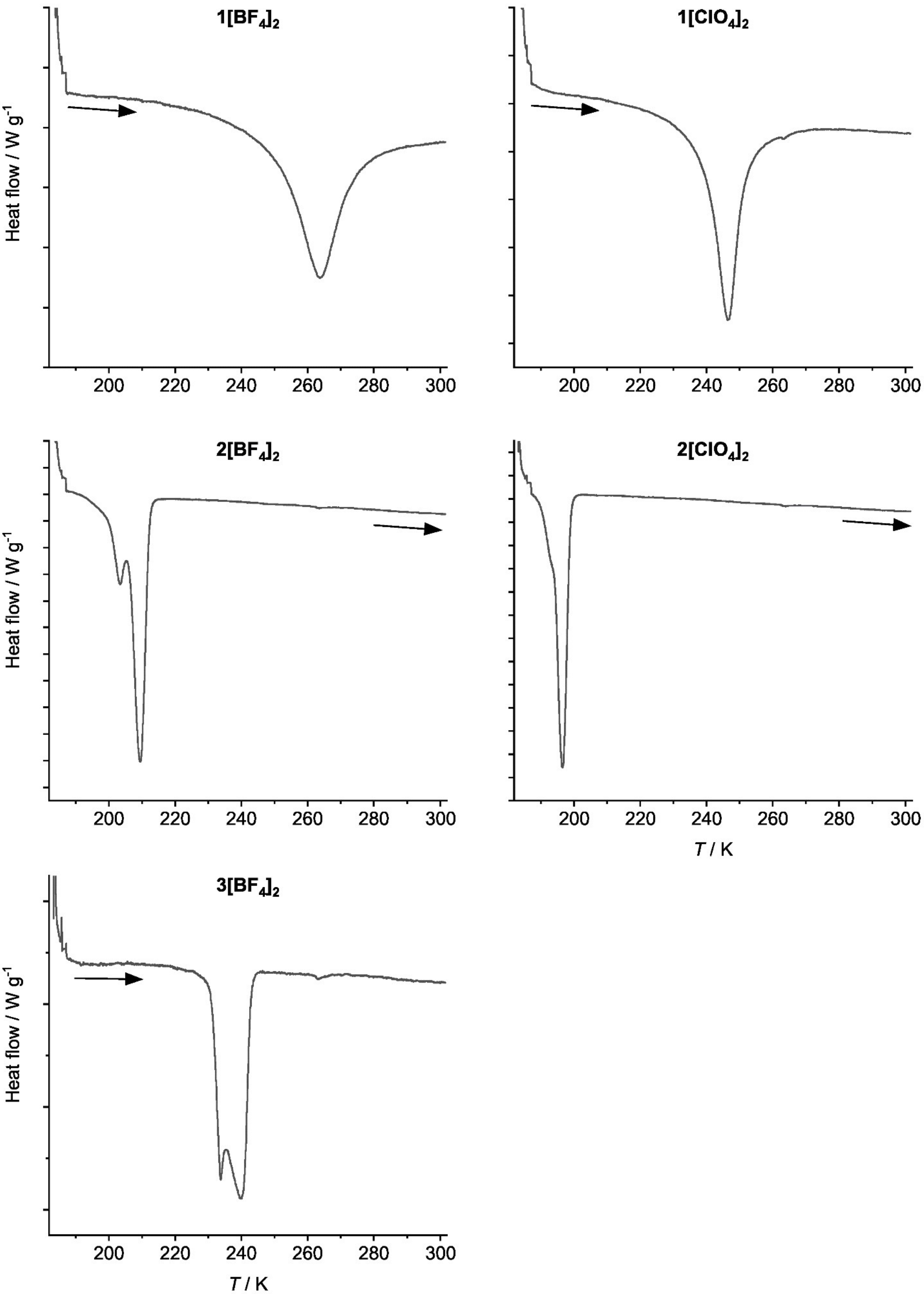

Figure S14 DSC measurements of the complexes in this work, measured in warming mode with a 5 K min^{-1} temperature ramp. These data are replotted in Figure S15, and in Figure 4 (main article).

Data for **3[ClO$_4$]$_2$** were not measured, because its high→low-spin transition temperature ($T_{½}$↓ = 158 K) is below the low-temperature limit of our calorimeter (183 K).

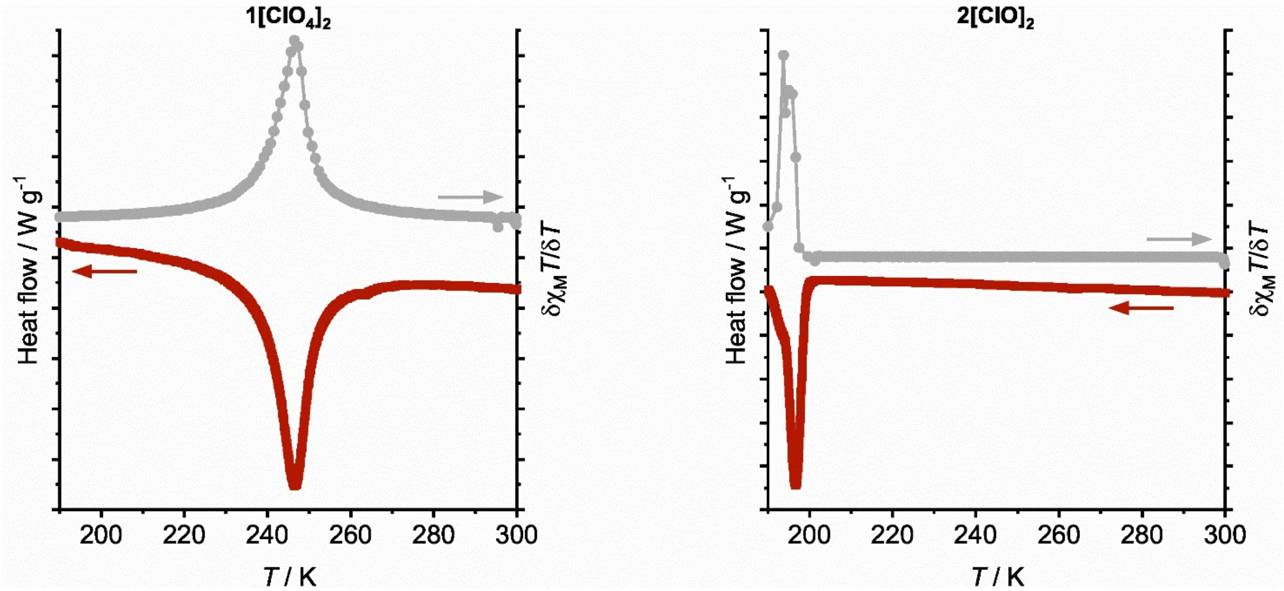

Figure S15 Comparison of the DSC data for the ClO_4^- salts in this work (red), with the first derivative of the magnetic data (grey; Figure 3, main article). All data were measured on a warming temperature ramp (the arrows indicate the relevant *y* axis scale for each plot).

Equivalent plots for the BF_4^- salts are in Figure 4 (main article).

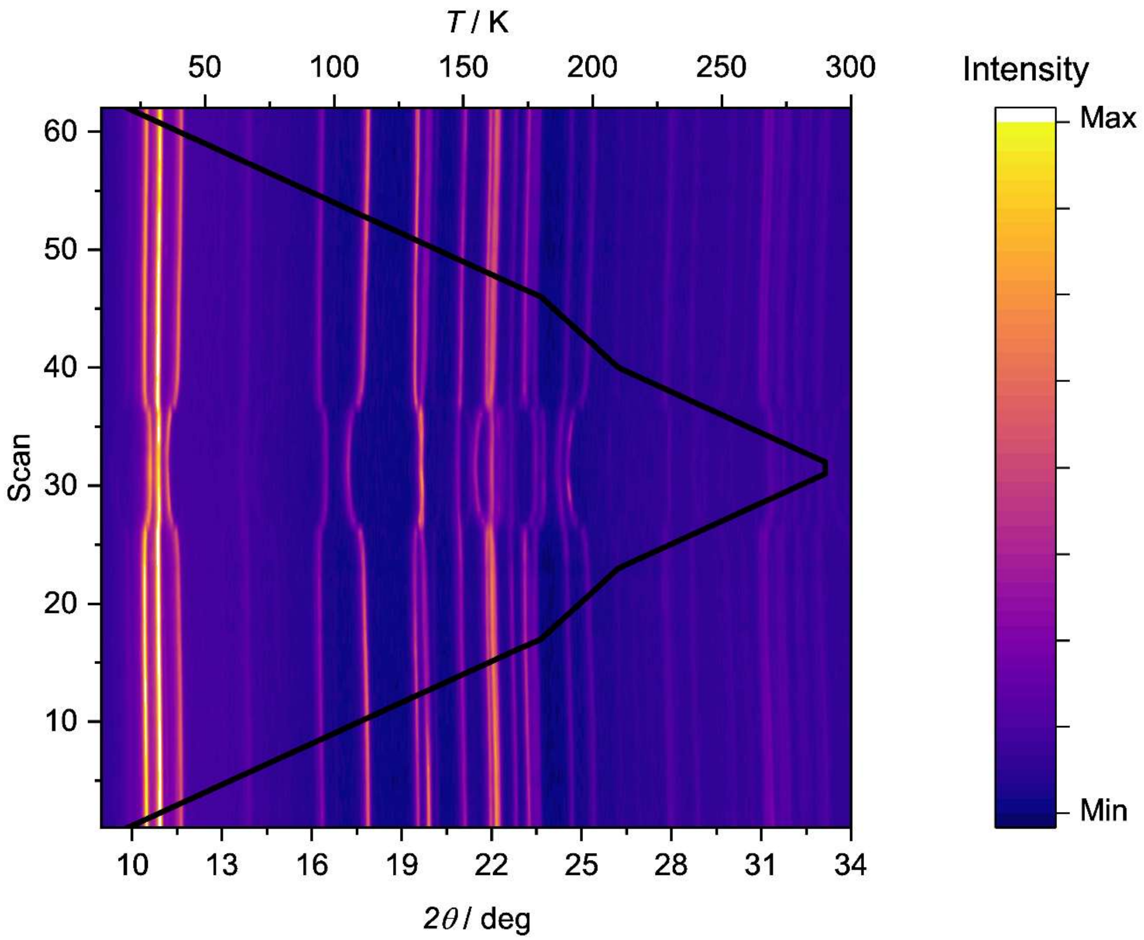

Figure S16 Variable temperature X-ray powder diffraction data for **1[ClO$_4$]$_2$**, showing the shifts of the Bragg peak positions associated with SCO. Data were collected on a 20-290-20 K temperature ramp at 10 K intervals, with additional data every 5 K between 180-210 K; the black line indicates the temperature of each scan, scaled against the top axis.

Corresponding data for **2[BF$_4$]$_2$** and **3[BF$_4$]$_2$** are shown in Figure 5 (main article).

Representative powder patterns at different temperatures from this experiment are shown in Figure S17, and these data are discussed further in the caption to that Figure on the next page.

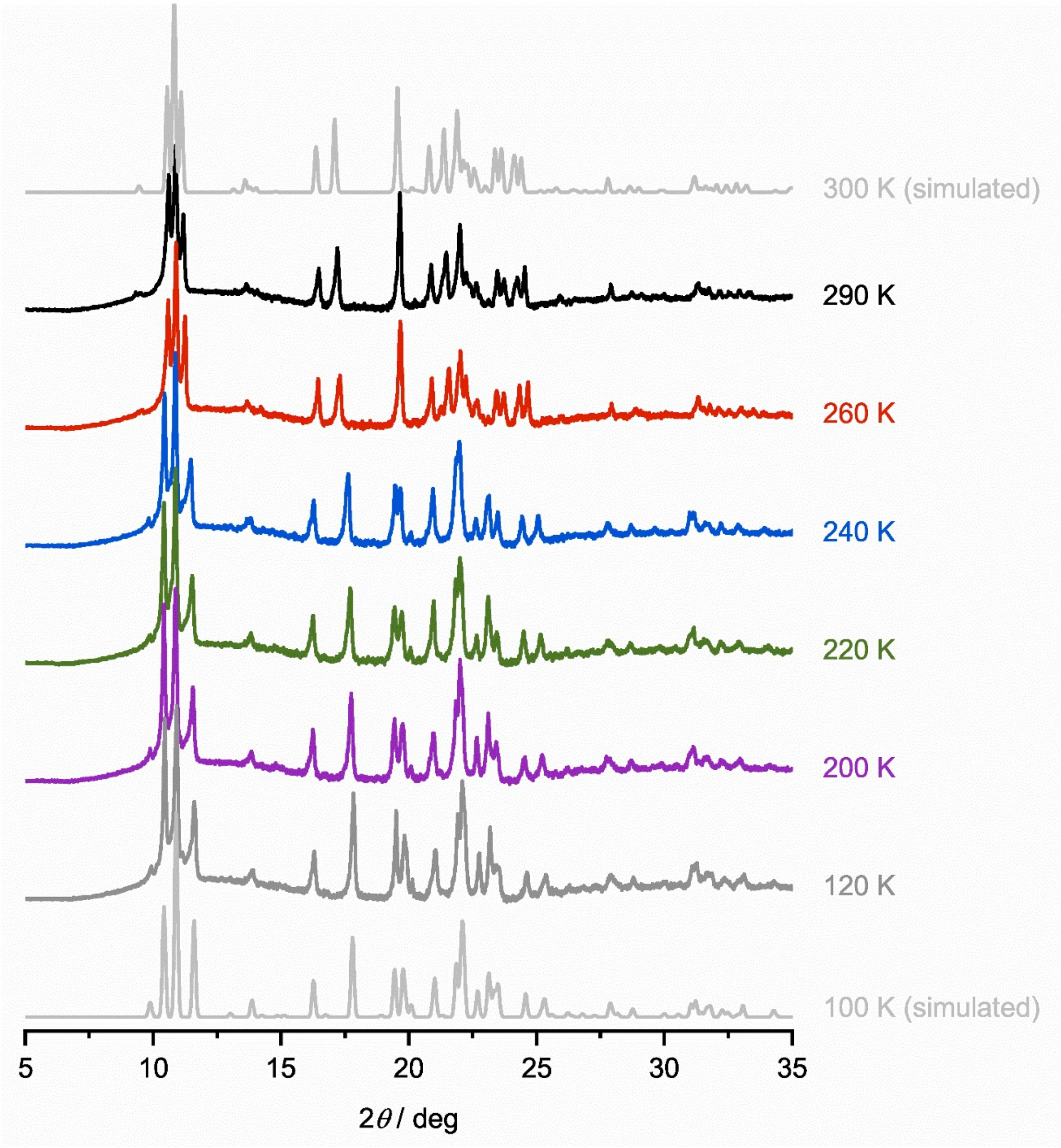

Figure S17 Representative variable temperature powder diffraction data from **1[ClO$_4$]$_2$**. Simulations of the high- and low-temperature data derived from the single crystal structures of the compound are also included. Data over the full temperature range in warming and cooling modes are plotted in Figure S16.

Unlike **2[BF$_4$]$_2$** and **3[BF$_4$]$_2$** (Figure 5, main article), **1[ClO$_4$]$_2$** undergoes a more gradual SCO equilibrium without hysteresis at $T_{½}$ = 245 K. The approximate width of the transition, defined as the temperature range between 20 % and 80 % completeness,[21] is between 235-250 K in the magnetic data (Figure 3, main article). Consistent with that, the Bragg peak positions evolve continuously as the transition progresses, which is most pronounced between *ca* 255 and 235 K – that is, at $T_{½}$ ±10 K (Figure S16).

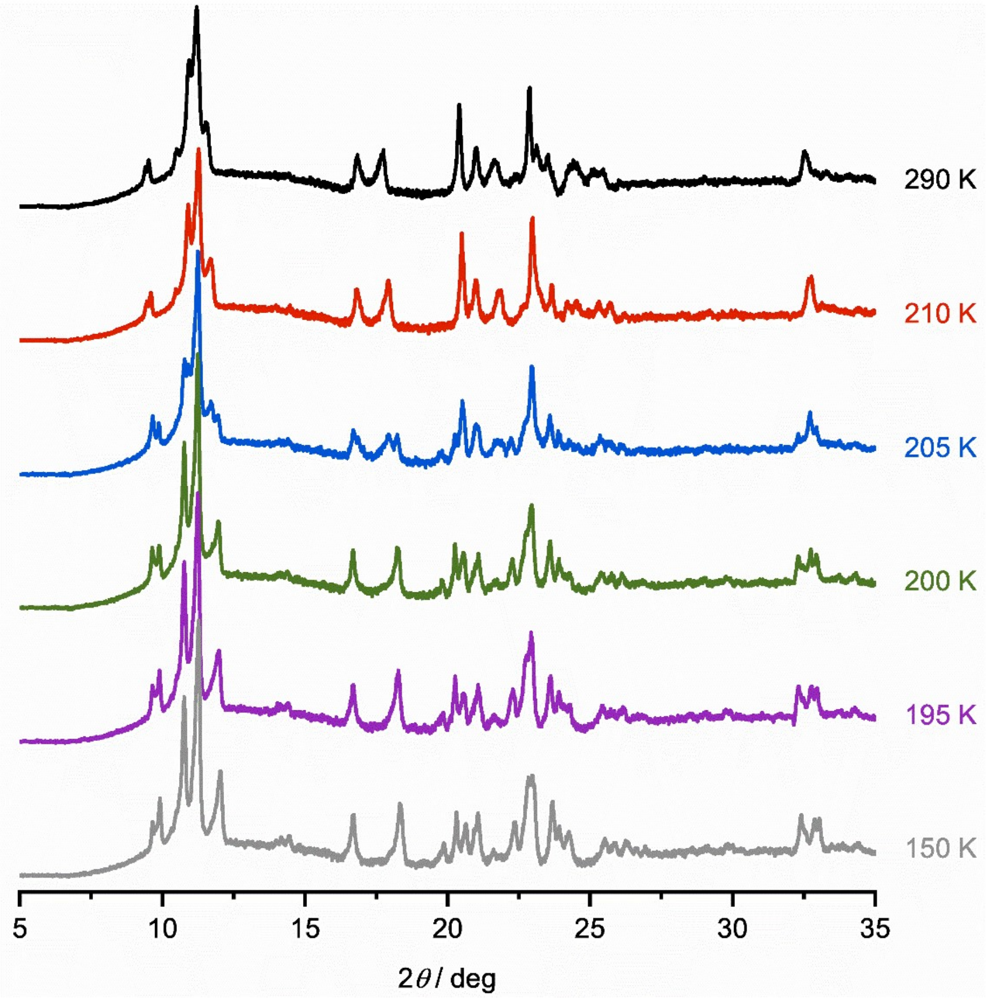

Figure S18 Representative variable temperature powder diffraction data from **2[BF$_4$]$_2$**, measured in cooling mode. Data over the full temperature range in warming and cooling modes are plotted in Figure 5 (main article).

The compound undergoes an abrupt spin-transition at $T_{½}$ = 201 K in its magnetic data (Figure 3, main article). This sample is high-spin at between 290-210 K, and low-spin at 200 K and below. At 205 K, the data show a mixture of distinct the high-spin and low-spin phases.

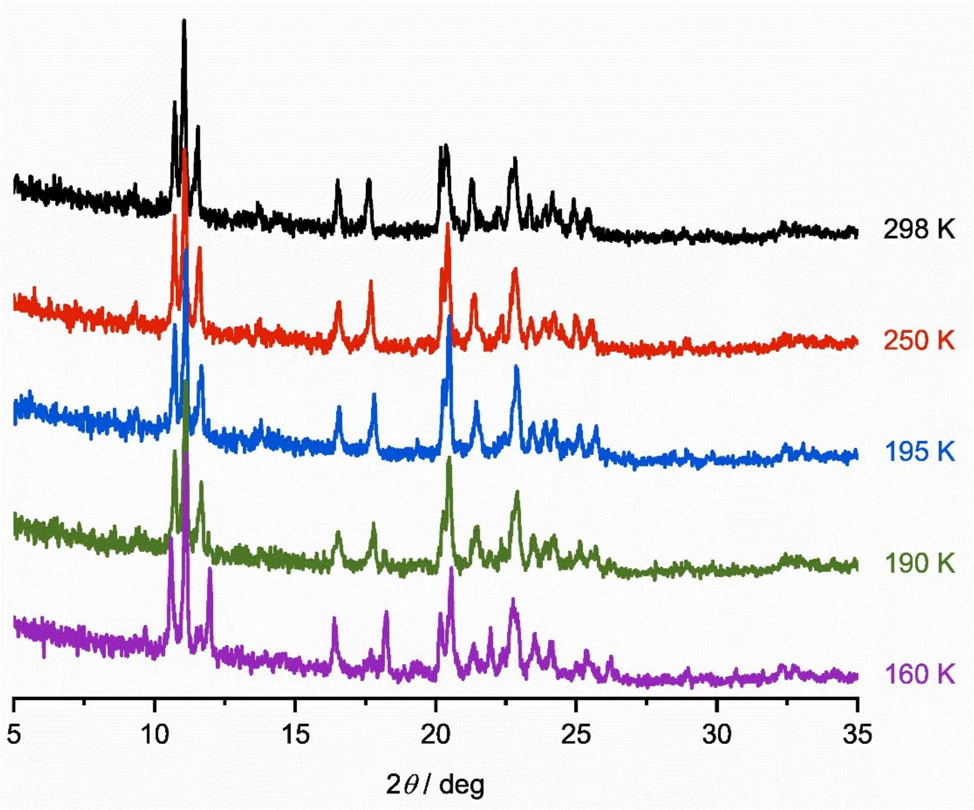

Figure S19 Representative variable temperature powder diffraction data from **2[ClO$_4$]$_2$**, measured in cooling mode.

These variable temperature data were measured on a different diffractometer from the other samples, with a more limited temperature range. Despite the sparser data, the sample behaves similarly to the other compounds investigated.

The compound undergoes an abrupt spin-transition with a narrow thermal hysteresis, at $T_{½}\downarrow$ = 190 K in cooling mode (Figure 3, main article). This sample is high-spin at between 298-195 K, and predominantly high-spin with a small low-spin fraction at 190 K. The compound is low-spin at 160 K.

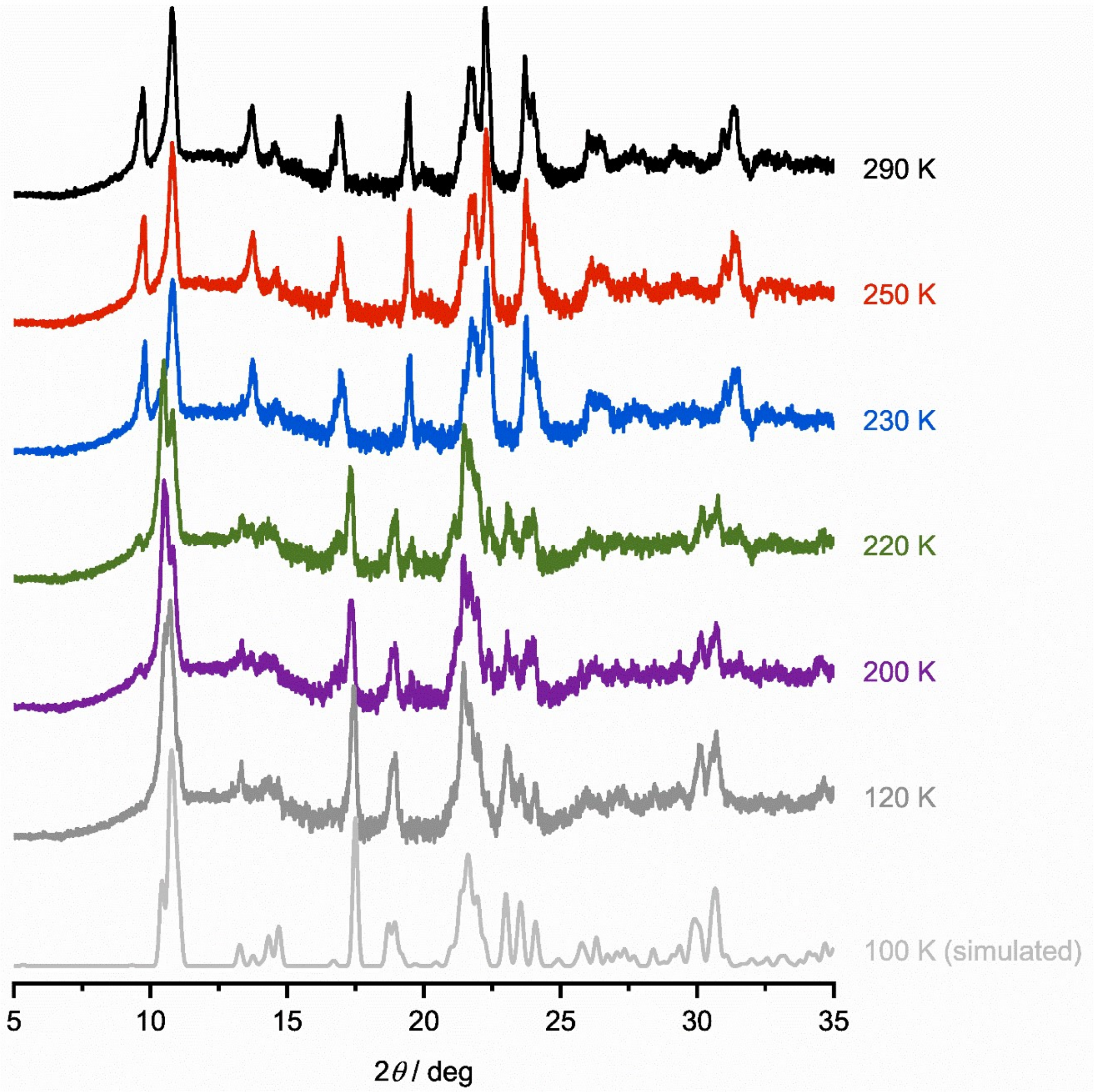

Figure S20 Representative variable temperature powder diffraction data from **3[BF$_4$]$_2$**, measured in cooling mode. A simulation of the low-temperature data derived from the single crystal structure of the compound is also included. Data over the full temperature range in warming and cooling modes are plotted in Figure 5 (main article).

The magnetic data for this compound show an abrupt, hysteretic spin-transition with at $T_{½}\downarrow$ = 228 K in cooling mode (Figure 3, main article). This sample is high-spin at between 290-230 K, and low-spin at 120 K. The data show a mixture of the high-spin and low-spin phases at 220 K while, unexpectedly, a small fraction of high-spin material is also evident at 200 K. This is discussed in the main article.

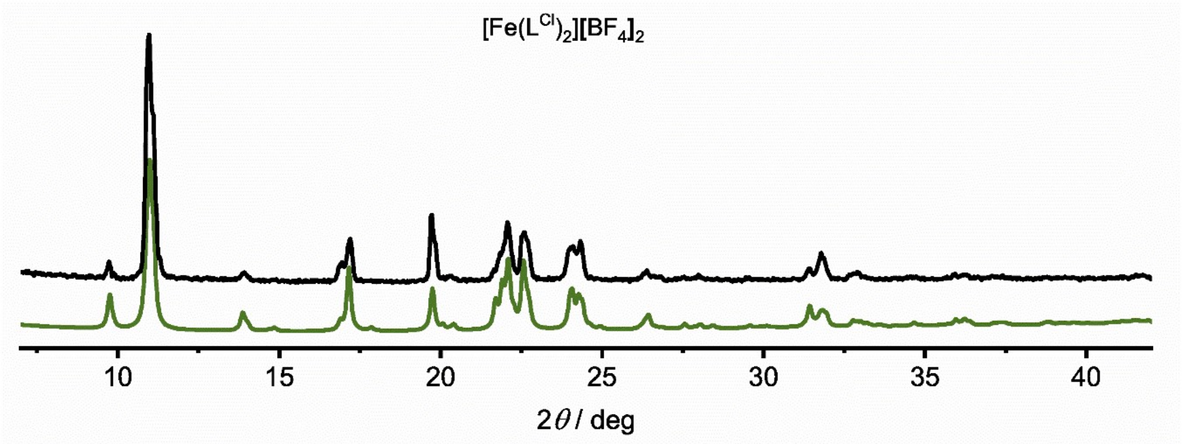

Figure S21 Measured (black) and simulated (green) room temperature X-ray powder diffraction data for $[Fe(L^{Cl})_2][BF_4]_2$. Fitted unit cell parameters from the simulation are in Table S5.

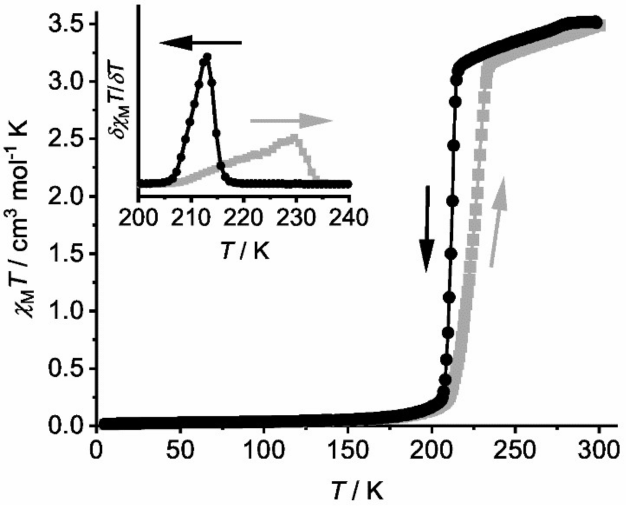

Figure S22 Variable temperature magnetic susceptibility data for $[Fe(L^{Cl})_2][BF_4]_2$, measured in cooling (black) and warming (grey) modes at a scan rate of 2 K min^{-1}. The inset shows the first derivative of the plot.

The spin-transition in $[Fe(L^{Cl})_2][BF_4]_2$ occurs at $T_{½}\downarrow$ = 212 and $T_{½}\uparrow$ = 224 K. It resembles **3[BF$_4$]$_2$** in having an abrupt cooling branch and a more gradual warming branch (Figure 3, main article), but occurs at 15 K higher temperature. That similarity is reasonable since the two samples are isomorphous by powder diffraction (Table S5).

However, the monotonic increase of $\chi_M T$ with T at higher temperatures is unusual and could be an experimental artefact. Unfortunately, it was not possible to repeat the measurement or characterise this material further during the timescale of the study. For that reason, these data are not presented as part of the main article.

References

1 L. Wang, N. Liu and B. Dai, *RSC Adv.*, 2015, **5**, 82097–82111.

2 I. Galadzhun, I. Capel Berdiell, N. Shahid and M. A. Halcrow, *CrystEngComm*, 2019, **21**, 6330–6334.

3 G. M. Sheldrick, *Acta Cryst. Sect. A: Found. Adv.*, 2015, **71**, 3–8.

4 G. M. Sheldrick, *Acta Cryst. Sect. C: Struct. Chem.,* 2015, **71**, 3–8.

5 L. J. Barbour, *J. Appl. Cryst.*, 2020, **53**, 1141–1146.

6 O. V. Dolomanov, L. J. Bourhis, R. J. Gildea, J. A. K. Howard and H. Puschmann, *J. Appl. Cryst.*, 2009, **42**, 39–341.

7 A. Coelho, *J. Appl. Cryst.*, 2018, **51**, 210–218.

8 C. J. O'Connor, *Prog. Inorg. Chem.*, 1982, **29**, 203–283.

9 P. Guionneau, M. Marchivie, G. Bravic, J.-F. Létard and D. Chasseau, *Top. Curr. Chem.*, 2004, **234**, 97–128.

10 I. Capel Berdiell, R. Kulmaczewski and M. A. Halcrow, *Inorg. Chem.*, 2017, **56**, 8817–8828.

11 J. K. McCusker, A. L. Rheingold and D. N. Hendrickson, *Inorg. Chem.*, 1996, **35**, 2100–2112.

12 M. A. Halcrow, *Chem. Soc. Rev.*, 2011, **40**, 4119–4142.

13 L. J. Kershaw Cook, R. Mohammed, G. Sherborne, T. D. Roberts, S. Alvarez and M. A. Halcrow, *Coord. Chem. Rev.*, 2015, **289–290**, 2–12.

14 J. M. Holland, J. A. McAllister, C. A. Kilner, M. Thornton-Pett, A. J. Bridgeman and M. A. Halcrow, *J. Chem. Soc. Dalton Trans.*, 2002, 548–554.

15 S. Vela, J. J. Novoa and J. Ribas-Arino, *Phys. Chem. Chem. Phys.*, 2014, **16**, 27012–27024.

16 I. Capel Berdiell, E. Michaels, O. Q. Munro and M. A. Halcrow, *Inorg. Chem.*, 2024, **63**, 2732–2744.

17 L. J. Kershaw Cook, F. L. Thorp-Greenwood, T. P. Comyn, O. Cespedes, G. Chastanet and M. A. Halcrow, *Inorg. Chem.*, 2015, **54**, 6319–6330.

18 N. Suryadevara, A. Mizuno, L. Spieker, S. Salamon, S. Sleziona, A. Maas, E. Pollmann, B. Heinrich, M. Schleberger, H. Wende, S. K. Kuppusamy and M. Ruben, *Chem. – Eur. J.*, 2022, **28**, e202103853.

19 I. Capel Berdiell, R. Kulmaczewski, N. Shahid, O. Cespedes and M. A. Halcrow, *Chem. Commun.*, 2021, **57**, 6566–6569.

20 R. Kulmaczewski, L. J. Kershaw Cook, C. M. Pask, O. Cespedes and M. A. Halcrow, *Cryst. Growth Des.*, 2022, **22**, 1960–1971.

21 M. Marchivie, P. Guionneau, J.-F. Létard and D. Chasseau, *Acta Cryst. Sect. B: Struct. Sci.*, 2003, **59**, 479–486.
